# Multielectrode recordings of cockroach antennal lobe neurons in response to temporal dynamics of odor concentrations

**DOI:** 10.1007/s00359-022-01605-7

**Published:** 2023-01-16

**Authors:** Harald Tichy, Alexander Martzok, Marlene Linhart, Lydia M. Zopf, Maria Hellwig

**Affiliations:** Department of Neurosciences and Developmental Biology, University of Vienna, Faculty of Life Sciences, Djerassiplatz 1, 1030 Vienna, Austria

**Keywords:** Electrophysiology, Tetrode, Cockroach, Olfaction, Antennal lobe

## Abstract

The initial representation of the instantaneous temporal information about food odor concentration in the primary olfactory center, the antennal lobe, was examined by simultaneously recording the activity of antagonistic ON and OFF neurons with 4-channel tetrodes. During presentation of pulse-like concentration changes, ON neurons encode the rapid concentration increase at pulse onset and the pulse duration, and OFF neurons the rapid concentration decrease at pulse offset and the duration of the pulse interval. A group of ON neurons establish a concentration-invariant representation of odor pulses. The responses of ON and OFF neurons to oscillating changes in odor concentration are determined by the rate of change in dependence on the duration of the oscillation period. By adjusting sensitivity for fluctuating concentrations, these neurons improve the representation of the rate of the changing concentration. In other ON and OFF neurons, the response to changing concentrations is invariant to large variations in the rate of change due to variations in the oscillation period, facilitating odor identification in the antennal-lobe. The independent processing of odor identity and the temporal dynamics of odor concentration may speed up processing time and improve behavioral performance associated with plume tracking, especially when the air is not moving.

## Introduction

Insects are able to use odor plumes with remarkable ease to navigate toward odor sources. The male cockroach, *Periplaneta americana,* tracking a plume of female pheromone navigates up-wind (positive anemotaxis) while in contact with the attractive odor (Willis and Avondet 2005; Willis et al. 2008; Willis 2008; Lockey and Willis 2015). When the wind flow is stopped, however, the cockroach continues to track the odor plume to its source, although it takes longer to locate it. Stopping the wind in the tunnel “leaves a slowly expanding plume hanging in a zero wind environment” (Willis et al. 2008), which implies that anemotaxis is not obligatorily used by male cockroaches when plume tracking. True chemotaxis in cockroaches is less well-known than in lobsters and crabs. Behavioral studies in an aquatic turbulent odor plume have shown that lobsters use a spatial gradient generated by the size and shape of odor pulses encountered in the plume to locate the source (Moore and Atema 1991; Basil and Atema 1994). The onset slopes and the closely correlated peak concentration of odor pulses increase with decreasing distance to the odor source and provide the strongest gradient that points in the direction of the odor source (Moore and Atema 1991; Atema 1995, 1996; Zettler and Atema 1999). Chemoreceptors of the spiny lobster, *Panulirus argus*, and the clawed lobster, *Homarus americanus*, function as “concentration slope” or “pulse slope detectors” which respond differently to a range of pulse onset slopes or rates of rising pulse concentration (Devine and Atema 1982; Atema 1985; Gomez and Atema 1996a, b; Zettler and Atema 1999; Zimmer-Faust et al. 1995; Marschall and Ache 1989; Derby et al. 2001).

Are “pulse slope detectors” unique to marine crustaceans or do they also exist in the cockroach? The cockroach’s peripheral olfactory system has been extensively studied (reviews: Boeckh and Ernst 1987; Boeckh et al. 1990), resulting in a fairly complete list of olfactory sensilla, their structures, innervation patterns and distributions on the antennae (Sass 1972, 1976, 1978; Altner et al. 1977, 1983; Toh 1977; Schaller 1978; Fujimura et al. 1991). Based on electrophysiological recordings, the responses of many ORNs to a repertoire of chemically pure organic odors and to natural food like the odor of lemon oil have been explored intensively. Lemon-oil odor contains many different compounds and elicits, when tested separately or as a mixture, strong excitatory responses in ORNs located in both basiconic and trichoid sensilla (Sass 1976, 1978; Selzer 1981). Although recognizing odor mixtures requires more neuronal resources compared to single compounds, modeling studies and physiological experiments revealed that complex odor mixtures evoke more robust and reliable activity patterns over a wide concentration range than single odors; they also generate a more efficient neural code (Chan et al. 2018). By testing slowly fluctuating changes in the concentration of the lemon-oil odor, we identified in a morphologically distinct trichoid sensillum (single-walled type C, Schaller 1978), two types of ORNs which display antagonistic ON and OFF responses. Increasing odor concentration raises impulse frequency in the ON ORN and lowers it in the OFF ORN. Correspondingly, contrary effects are produced by decreasing odor concentration (Hinterwirth et al. 2004; Tichy et al. 2005; Burgstaller and Tichy 2011, 2012; Hellwig and Tichy 2016; Tichy and Hellwig 2018).

The ON and OFF ORNs are highly sensitive to two independent components of the olfactory stimulus: the instantaneous odor concentration and its rate of change. Furthermore, sensitivity for both components are modulated by fluctuating changes in odor concentration. During brief oscillations with rapid concentration changes, both types of ORNs improve the gain for odor concentration and reduce the gain for the rate of concentration change. Conversely, when odor concentration oscillates slowly with long periods, the gain for the rate of change increases at the expense of the gain for concentration. Reducing gain for the concentration rate at brief oscillation periods protects against saturation, whereas increasing gain for the concentration rate at long oscillation periods maximizes the detection of slow concentration changes (Tichy and Hellwig 2018). This dynamic gain control of the ON and OFF ORNs favors a function as “pulse slope detectors”. A basic prerequisite for such a function is that the responses to changes in odor concentration are invariant to the air flow velocity. When the flow velocity increases, the amount of air volume flowing across an area per unit of time increases. Thereby, the number of molecules arriving per unit time at a unit antennal surface also increases. A recent study under slowly changing concentrations and at different flow rates has shown that the ON and OFF ORNs on the cockroach’s antenna do not respond to the molecule arrival rate but to the actual rate of concentration change independently of air velocity (Hellwig et al. 2019).

Excitatory responses to concentration pulses of lemon-oil odor were also observed in ORNs located in two types of basiconic sensilla (single-walled type A and B; Schaller 1978). When odor concentration fluctuates, however, the sensitivity for the instantaneous concentration and the rate of concentration change is invariant to the duration of the oscillation period: shallow concentration waves provided by long periods has the same effect to the response to instantaneous concentration as steep concentration waves at brief periods (Tichy et al. 2020a). This contrasts with the ability for gain control of the ON and OFF ORNs in trichoid sensilla which makes them particularly well-suited for encoding temporal information inherent in the olfactory signal. The high sensitivity of ORNs in basiconic sensilla over a range of concentrations rates facilitates the encoding of odor identity (Tichy et al. 2020a). The presence of food-odor responsive ORNs in both basiconic and trichoid sensilla may be less an adaptation to increase the sensitivity to particular odor mixtures than to extract and process different aspects of the food odor in parallel.

None of the numerous studies on the antennal lobe of insects have reported using continuous concentration changes whose rates were defined from instant-by-instant in terms of change in concentration over time and, moreover, systematically varied. Accordingly, the work reported here is an effort to outline the behavior of cockroach’s antennal-lobe neurons upon both transient pulse-like and slowly oscillating concentration changes. The goal is to better understand the basic principles underlying the encoding of the instantaneous concentration and the rate of this change. The ON and OFF ORNs play an important but perhaps not exclusive role in providing excitatory signals in response to increments and decrements, respectively, in the concentration of the lemon-oil odor. These antagonists are always present in pairs in trichoid sensilla, making up 6% (~ 4400) of the total sensillum number on the antenna (Schaller 1978; Altner et al. 1983). This low sensory input from the ON and OFF ORNs to the antennal-lobe, the primary olfactory center, makes synaptic connection with two types of neurons, namely, the postsynaptic principle neurons, termed projection neurons, which provide olfactory information to higher order neuropils via parallel pathways, and the mostly inhibitory local interneurons, which shape the transmission from ORNs to projection neurons (Boeckh and Ernst 1987; Distler and Boeckh 1997a, b; Nishino et al. 2012; Watanabe et al. 2017; Paoli et al. 2020; Fuscà and Kloppenburg 2021).

The present study examined the transfer and representation of information about physical and temporal properties of a food odor stimulus beyond its chemical identity. We used self-made tetrodes consisting of four microwires to tap into the neural signals in the antennal lobe. This type of electrode array allowed us to differentiate among multiple, simultaneously recorded close-by neurons. We recorded ON and OFF neurons together and at the same time with a single tetrode, we tested concentration pulses of varying amplitude and oscillating concentration changes of varying period, we evaluated the neurons response gain for both kinds of concentration changes, and we asked whether gain is controlled with respect to the input signal variations. Are particular features of the odor stimulus such as concentration emphasized while neglecting the rate of change, or is a high gain for the rate of change traded for a low gain for the instantaneous concentration? Is odor identity transformed into a representation that is robust to changes in concentration or the rate of change? Is the time scale of the responses altered and adaptation accentuated, or has the slope of the input–output been changed? The results show what temporal features of the odor signal from a dynamically complex odor signal are represented at the level of the antennal lobe and potentially available for plume tracking behavior.

## Materials and methods

### Experimental animals

*Periplaneta americana* were reared in a 12:12 h light–dark cycle at 28 °C and more than 70% relative humidity. Oat flakes and water were offered ad libitum*.*

### Intact brain preparation

Adult male cockroaches were anesthetized by CO_2_ and placed in a self-built Perspex holder. The head was sealed around with low-melting dental wax and the thorax and abdomen were secured with strips of Parafilm draped around the holder. The antennae were fastened with adhesive tape and dental cement in a shallow groove of a Perspex stage projecting from the holder. The head capsule was opened by cutting a window between the two compound eyes and the base of the antennae. Tracheae, pharynx and the sheath overlying one antennal lobe were partially removed with fine forceps. The preparation was then supplied with physiological saline solution (in mM: 130 NaCl, 12 KCl, 6 CaCl, 3 MgCl, 23 glucose, and 4 HEPES) for the duration of the experiment. A silver chloride reference electrode was inserted into the contralateral compound eye.

### Odor stimulation

Lemon oil is a very effective odor in eliciting activity from antennal ORNs and antennal lobe neurons (Boeckh 1974; Sass 1978; Selzer 1981, 1984). It contains different compounds of several chemical classes (Günther 1968; Shaw 1979). The sensory consistency of natural fruits can differ greatly depending upon the regional diversity, ripeness stage, and storage. Therefore, synthetic lemon oil (relative density = 0.85, Art. 5213.1; Carl Roth GmbH + Co. KG; Karlsruhe, Germany) was used as a standardized fruit odor stimulus.

The air dilution flow olfactometer used to deliver the odor stimulus was described recently (Tichy et al. 2020b). In brief, the stimulation technique is as follows. Clean compressed air was divided into two streams with equal flow rates. One stream flowed through a tank with the undiluted lemon oil. The other stream was led through an empty tank of the same design and remained clean. Then, the two air streams passed through electrical proportional valves and electronic flow meters. The two streams were then combined. A 180 degree phase shift of the valves’ control voltages (digital analog outputs of CEDmicro 1401mkII) ensured that the total flow rate of the combined air stream was held constant at 1.5 m/s as the flow rate ratio of the odor-saturated to clean air varied. This ratio was regulated by means of the output sequencer function of the data acquisition software (Spike2, v.8), using a self-written sequencer script. The mixed air stream emerged from a nozzle 7 mm in diameter at a distance of 10 mm from the antenna. A suction tube continually removed the air around the antenna. The digitized output voltage of the electronic flow meters, calibrated by the manufacturer for flow rate, was used to monitor the flow profiles of the two individual air streams and of the mixed air stream representing the odor delivery during stimulation. The concentration of the stimulus was determined by the flow rate ratio of odor-saturated air to clean air and expressed as percentages of the total flow rate: “0%” means clean air only and indicates that the air stream directed onto the cockroach does not contain the odor of lemon oil; “50%” means odorized and clean air streams are mixed in a 50:50 ratio. A photoionization detector (200A miniPID, Aurora Scientific) was used to verify the time course of slow concentration changes.

### Tetrode recording setup

**Fig. 1:**
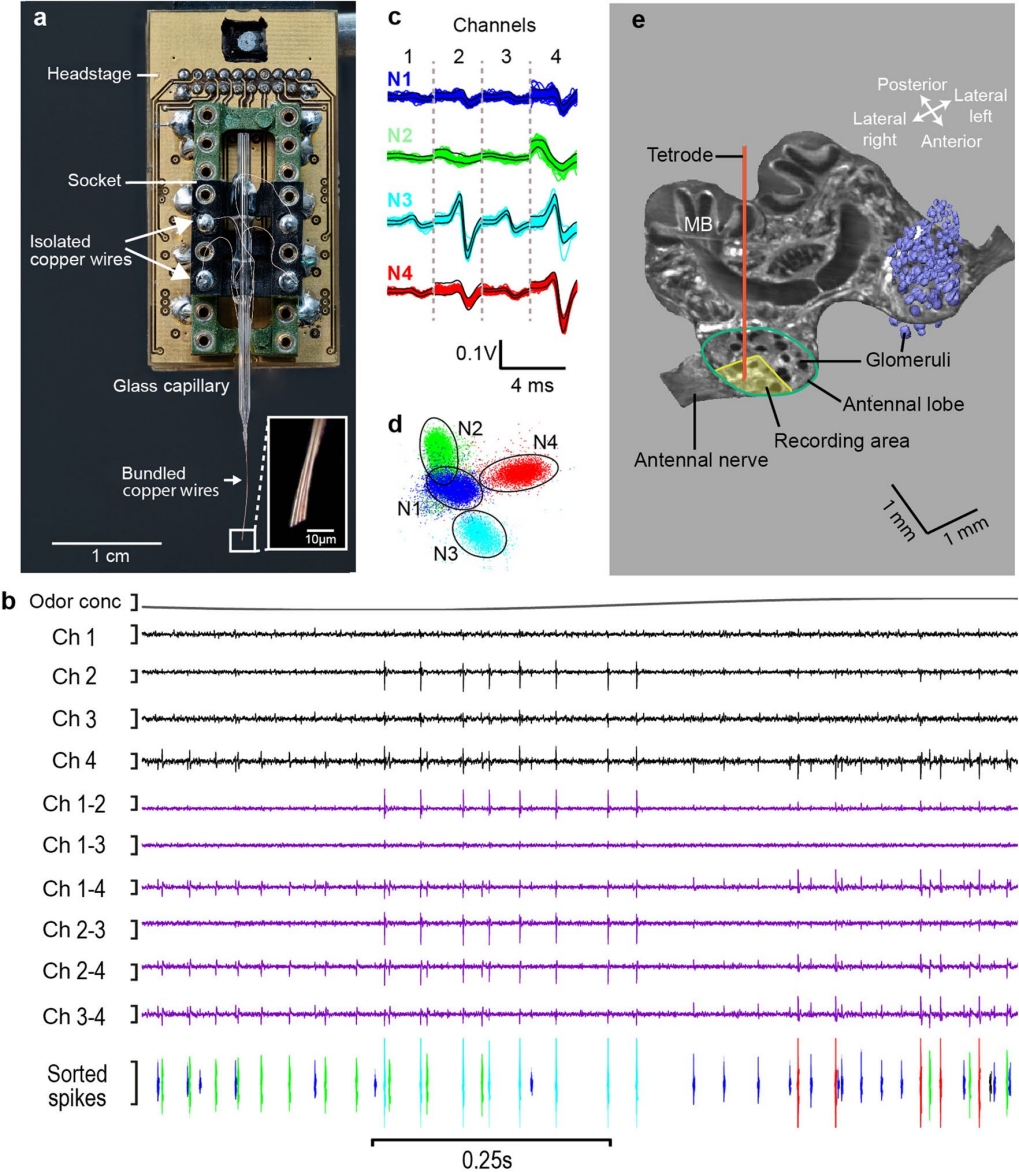
Tetrode recordings from antennal lobe neurons using copper wire electrodes. **a** Endings of four copper wires are soldered to four separate lugs of a pin connector (black) and plugged onto the head stage amplifier (green). On the other end, the micro wires are waxed together, attached on a glass capillary and glued on the socket of the pin connector; the ends of the wires are cut off (insert). **b** Oscillating change of the lemon-oil odor concentration (period duration is 3 s) elicit neuronal response in the antennal lobe recorded with the tetrode. The uppermost trace shows the time course of odor concentration (scale bare: 0–100%). The four black traces represent the electrical signals obtained in the four recording channels (scale bare: 0.1 V). The closer the recorded neurons are positioned to the electrode, the stronger and better signal to noise ratios can be achieved. The purple channels provide the pairwise differentiated neuronal response of the four recording channels (scale bare: 0.1 V), reducing noise and verifying that the source of the signal is closely related to the position of the electrode tip. The lowermost trace shows the color coded spike shapes of four sorted, color coded units associated with four single neurons. **c** Superimposed spike waveform templates of the four color-coded neurons (N1–N4) drawn for each tetrode channel next to each other; vertical dashed lines separate the templates. **d** Feature vectors from spike waveforms, extracted and computed for each channel by principal component analysis, isolate four dense clusters of the spiking events of the four neurons. The ellipsoidal boundaries of the four clusters show almost no overlapping. **e** 2D HREM image of a horizontal section through the cockroach’s brain visualizes the antennal nerve, the antennal lobe and the schematic positioning of the electrodes during tetrode recording. A 3D reconstruction of the glomeruli of the lateral left antennal lobe was produced from the stack of successive optical sections using AMIRA (V 6.0, Thermo Fisher Scientific)

Three close-by electrodes have proven to be a valuable tool to record multiple projection neurons in the two olfactory tracts connecting the antennal lobe with the mushroom bodies and the lateral horn in the honeybee (Brill et al. 2013, 2014). The key advantage is that the differential amplitude and waveform of action potentials recorded simultaneously on different wires forming an electrode array provide better sorting quality of individual units compared to single wire recordings. We constructed tetrodes to reliably isolate single unit activity in the cockroach antennal lobe, which is composed of densely packed neuropils. The tetrodes consisted of four insulated copper wires (15 µm diameters; Elektrisola) that were hold together with low melting wax (Polarit^®^ W 46, TH. C. Tromm GmbH) and then glued to a glass capillary that was fixed on a pin connector (Fig. 1a). The tetrode tips were cut with a razor blade at a 45 degree angle, so that their tip distances would not be too small to get a good tetrode effect (Fig. 1a, inset). The impedance (1–2 MΩ at 1 kHz) and quality of each electrode was tested by a nanoZ (White Matter LLC). The free ends of the electrodes were soldered to the pin connector, which was plugged into the socket of the head-stage amplifier (NPI Electronic Instruments) and fixed on a micromanipulator to achieve precise placement. The silver chloride wire inserted into the contralateral compound eye was connected to the reference electrode input of the head stage. The head-stage output was connected to a differential multichannel amplifier (DPA-2FL, NPI Electronic Instruments) which compared the recording with each of the four tetrode wires to the reference electrode, featuring four close spaced recording channels. The neural activity was also measured differentially from all pairwise combinations of the four recording channels (Fig. 1b). The signals from the tetrode wires were amplified, bandpass-filtered (0.1–3 kHz), and passed through a CEDmicro 1401mkII (Cambridge Electronic Design, 16 bit, 500 kHz) interface connected to a PC for online recording (sampling rate 20 kHz). Spike detection and spike sorting into clusters of distinct waveform features, peak amplitude or peak-to-mean amplitude ratio were performed offline using well-established commercial software (Spike2 v.8) (Fig. 1b, c) involving a Principal Component Analysis (Fig. 1d).

The tetrode was gently inserted into the frontal aspect of the antennal lobe which conforms to the projection region of the ON and OFF ORNs located in the trichoid sensilla as shown by Watanabe et al. (2017). A high resolution motorized micromanipulator (WPI HS6-3) slowly advanced the tetrode into the AL (depth 200–350 µm). Multi-unit activities were usually encountered and, if spike activities were observed with high signal-to-noise ratio on more than one electrode channel, an attempt was made to present the series of predetermined stimuli.

A High Resolution Episcopic Microscopy (HREM) image of the horizontal section of the cockroach’s brain illustrates the recording area of the antennal lobe at the entrance of the antennal nerve and the positioning of the electrode (Fig. 1e).

### Data collection

Extracellular tetrode recordings prevented the morphological identification of the recorded neurons, but allowed the analysis of individual-neuron’s responses to the same stimulus, yielding a robust classification to dynamic changes in odor concentrations. Moreover, the results enabled not only single neuron analysis but also an approximation of the information conveyed by the pooled responses. In 21 tetrode recording sessions, a total of 70 single units were isolated whose spike waveforms were consistent and reproducible for a given electrode and correspond to single neurons. The number of neurons isolated from each tetrode ensemble recorded in the antennal lobe ranged from 2 to 4, with an average of 3.4 ± 6.5 neurons/tetrode. Two major classes of lemon-oil odor neurons emerged: ON neurons (*n* = 23) that responded with an increase in activity to rising lemon-oil odor concentration, and OFF neurons (*n* = 18) that increased activity to falling lemon-oil odor concentration. In addition, however, there were other neurons (*n* = 29) which appeared to exhibit features of (1) ON neurons (*n* = 8), but they did not respond to low-amplitude concentration jumps and oscillating concentration changes during long periods, (2) OFF neurons (*n* = 12) which did not respond to low-amplitude concentration drops and concentration oscillation of long periods, and (3) neurons (*n* = 9) which discharged impulses spontaneously and maintained their frequency level during stimulation with the lemon-oil odor. These neurons were not included in further analysis.

The ON and OFF neurons were identified by presenting pulse-like concentration changes with sharp on and off edges. Jumps and drops in lemon-oil odor concentration from clean air to almost saturated and then back to clean air enabled the reliable detection of changes in the neuron discharge rate. By rapidly passing the threshold of excitation, a change in the discharge rate prevails over neural noise. Data for stimulus–response functions were obtained by running 3 series of 7 pulses with descending amplitudes from 97 to 18%. The pulse duration was 3 s and the interval between the onset of successive pulses was 10 s. The time elapsed between two successive pulses was the conditioning period of ON neurons for concentration jumps, and the duration of the concentration pulse was the conditioning period of OFF neurons for concentration drops. Therefore, the conditioning period of the ON neurons was three times longer than that of the OFF neurons. Short conditioning periods may reduce the subsequent magnitude of the response to transient concentration changes. Furthermore, the level of the conditioning concentration for the ON-neuron’s responses to concentration jumps was 0% throughout the experiments, but varied for the OFF-neuron’s responses to concentration drops according to the pulse concentration. These differences in both the conditioning periods and conditioning levels may lead to bias the identification of OFF neurons highly sensitive for drops and oscillations in the lemon-oil concentration. This may explain why the number of OFF neurons identified and analysed is smaller than that of the ON neurons. However, longer conditioning periods of the OFF neurons for concentration drops may place a limit on the number of responses which could be obtained from a given tetrode recording.

Note that the concentration measurements apply directly to the air stream used to produce the concentration changes and less directly to the olfactory sensillum on the antenna or even the receptive sites of the ORNs inside the sensillum. The possibility to assign instantaneous concentration values to the receptive sites is given only when the rate of change is slow. This ensures that the neurons’ impulse frequency can be correlated with instantaneous concentration values at the receptive sites during concentration changes and also correlated with accurate values for the rate of concentration change. Oscillating concentration changes allow changing the instantaneous concentration independently of the rate of change simply by varying the oscillation period. This offers an approach for investigating the relative degree to which the two parameters of the odor stimulus determine the ON and OFF neurons’ activity and to describe phase relationships. Seven different periods ranging from 1 to 120 s were tested at least three times in each experiment, and the change from one period to the next was continuous. The oscillation cycles covered a concentration range of roughly 90% between 5 and 95%, and hence the rate of concentration change ranged from ± 2%/s during the 120-s oscillation period to ± 230%/s during the 1-s oscillation periods.

### Statistical analyses

Statistical analyses and plots were made using SigmaPlot 10.0 software (Systat, Inc., San Jose, CA, USA). Least-squares linear and multiple regressions were used to assess the relationship between one or more independent variables of the odor stimulus (amplitude of the concentration pulse, instantaneous concentration, rate of concentration change) and the dependent variables (impulse frequency) (Burgstaller and Tichy 2011, 2012; Hellwig and Tichy 2016; Tichy et al. 2020a). The equation of the best fitting regression line used to approximate the relation between impulse frequency (*F*) and pulse concentration (Δ%) is *F* = *y*0 + *a* Δ*C*, where *y*0 is the height or the y-intercept of the regression line and *a* is the slope of the regression line indicating the gain of the neuron’s response, which is the change in impulse per second (imp/s) per unit change in pulse concentration (Δ%). The equation of the best fitting multiple regression used to approximate the relation between impulse frequency (*F*) and the instantaneous concentration (%) and its rate of change (%/s) is *F* = *y*0 + *a* d*C*/d*t* + *bC*, where *y*0 is the height or the *y*-intercept of the regression plane, the *b-*slope is the gain for the instantaneous odor concentration, indicating the change in impulse per second (imp/s) per unit change in concentration (%), and the *a-*slope is the gain for the rate of concentration change, indicating the change in impulse per second (imp/s) per unit change in the rate of change (%/s). *R*^2^ is the coefficient of determination. It measures what percentage of variation in impulse frequency is explained by the regression model or how tightly the frequency values are clustered around the regression. The *p* value tests the null hypothesis that a coefficient of the regression model (slope value) is equal to zero. The *p* value also indicates if there is a significant relationship between the impulse frequency (dependent variable) and the independent stimulus variables (amplitude of the concentration pulse, instantaneous concentration, rate of concentration change) described by the regression model (the slope is not equal to zero).

## Results

**Fig. 2:**
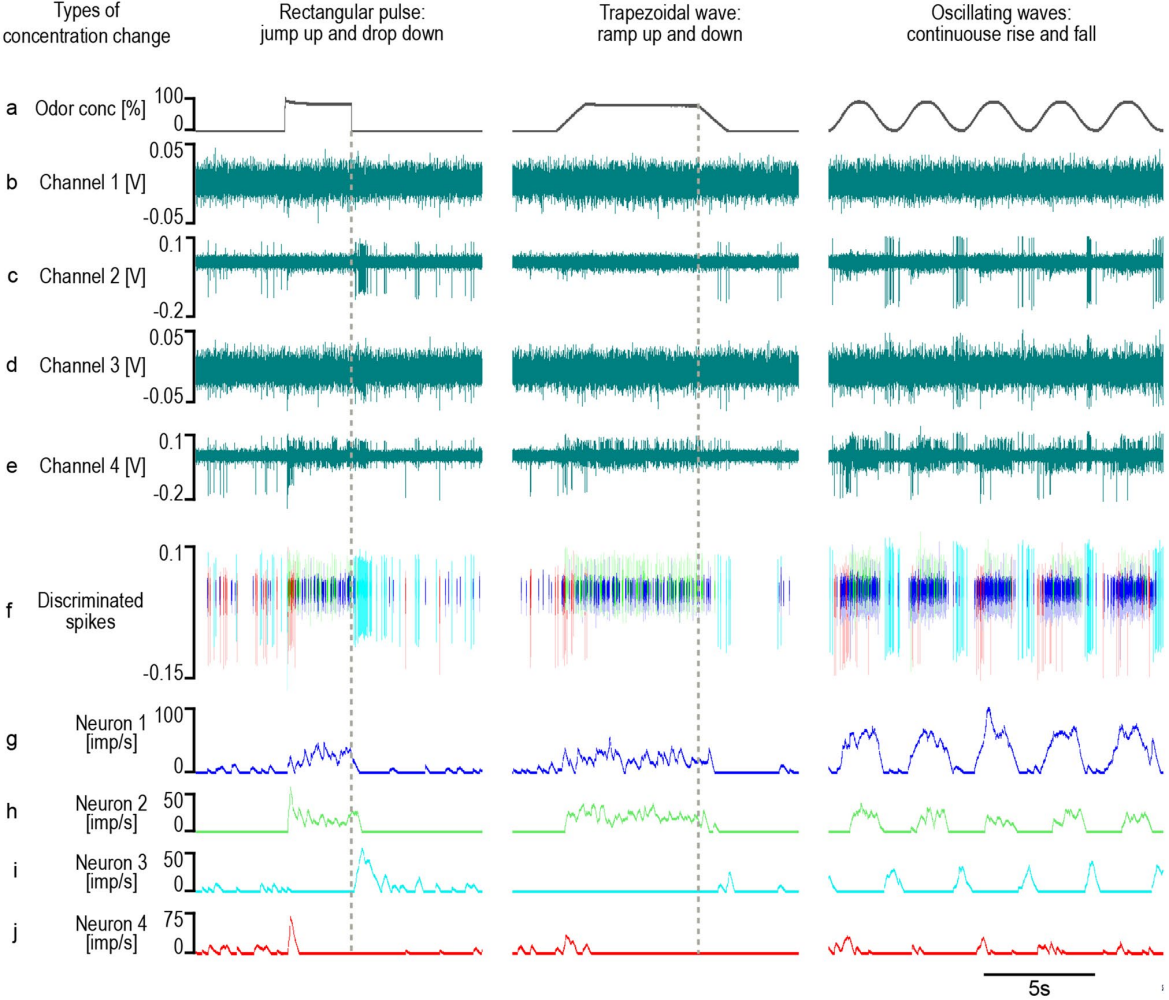
Example of an extracellular tetrode recording from four AL neurons responding to different dynamic changes in the concentration of the odor of lemon oil: a rectangular pulse, a trapezoidal wave and five sinusoidal waves. **a** Left column: time course of odor concentration illustrating a concentration jump from zero to 100%, followed 3 s later by a concentration drop back to the initial zero concentration. Middle column: time course of a 1-s ramp up from zero to 100% at a rate of + 100%/s, followed 5 s later by a 1-s ramp down to zero concentration at a rate of − 100%/s. Right column: time course of five continuous up and down cycles of sinusoidal concentration changes with a period of 3 s and at a constant amplitude of 100%, generating upward maximum rates of + 150%/s and downward maximum rates of − 150%/s. The negative values represent the downward direction of the concentration change. **b**–**e** Raw traces of the four tetrode channels 1 to 4 recorded at 20 kHz with bandpass filters at 0.1–3 kHz. Each microelectrode captures the activity of different neurons, so that overlapping spikes cannot be trivially resolved by detecting individual neurons in a distinct electrode, as in channels 1 and 3. **f** Sorting algorithm assigns the spikes to four single neurons which generate them; superimposed, color-coded activities of the four neurons in response to the different forms of concentration changes. **g**–**j** Response of the four neurons presented as time histogram (bin width, 200 ms) indicating that each neuron accentuates a different aspect of the changing concentrations. **g** ON-neuron 1 produces moderate activities for the duration of both the concentration pulse and the trapezoidal concentration wave, but strong responses during the up cycles of sinusoidal concentration waves. **h** ON-neuron 2 generates a phasic response to the concentration jump, a sustained discharge at low rates during the duration of both the concentration pulse and the trapezoidal concentration wave, and also produces sustained firing rates during the up cycles of the sinusoidal concentration waves. **i** OFF-neuron 3 generates a strong excitatory response to the concentration drop that appears at the pulse off, a weaker response at the end of the ramp down and stronger responses during the down cycles of the sinusoidal concentration wave. **j** ON-neuron 4 produces a moderate phasic response to the concentration jump, a weaker response during the ramp up and also weak responses during the up-cycles of the concentration waves. Pulse repetition intervals, 10 s; trapezoidal wave intervals, 10 s; V voltage

A typical example of a tetrode recording is shown in Fig. 2. Three different dynamic concentration changes in the 0–100% range were tested: a rectangular pulse with sharp on and off edges causing a jump and drop in concentration, a slow ramp up and ramp down forming the slopes of a trapezoidal concentration change, and slow oscillations with continuously rising and falling concentrations. Action potentials of varying amplitudes are evident in channels 2 and 4 (Fig. 2b–e). The recordings in channels 1 and 3 contain an amount of background noise impeding the detection and discrimination of weak spikes. In Fig. 2f, the superimposed, scaled and processed channel traces revealed the activity of four single neurons. A visual interpretation of their responses is provided by time histograms (Fig. 2g–j). There are three different ON neurons (Fig. 2g, h, j) with respect to the response rate as a function of time, and one OFF neuron (Fig. 2i). ON-neuron 1 (Fig. 2g) had a spontaneous discharge before stimulation that increased rapidly in response to both the concentration pulse and the trapezoidal concentration wave. During the up cycles of the sinusoidal concentration waves, the ON-neuron produced strong responses that consist of irregular, continuous discharges of action potentials without bunching or bursting of impulses. The faster rate of change during the concentration jump elicited lower impulse frequencies than the slower rates during oscillating concentration changes. ON-neuron 2 (Fig. 2h) was silent during the stimulus intervals and had a strong phasic response to the concentration jump, followed by a sustained low firing rate which outlasts the pulse, and then stops with a moderate discharge increase. The discharge was irregular but continuous during both the trapezoidal concentration wave and the up cycles of the concentration sinus. During the down cycles the ON-neuron became silent. OFF-neuron 3 (Fig. 2i) exhibited a low spontaneous discharge during the stimulus intervals that was reduced or absent during the odor stimulus. A strong excitatory response appeared as the concentration drop approached zero concentration, and a weaker one as the ramp down approached zero concentration, but during the down cycles of the sinusoidal concentration change a rapid peak discharge occurred. The faster rate of change during the concentration drop led to a stronger response than the slower rates during oscillating concentration changes. No activity was observed during the up cycles. ON-neuron 4 (Fig. 2j) had a spontaneous impulse discharge, a strong phasic response to the concentration jump, and moderate phasic responses to both the ramp up and the up cycles of the oscillations. The ON-neuron was not active during the down cycles.

The four neurons convey more information than merely the presence and absence of the odor of lemon oil, the concentration amplitude, the direction of the concentration change, the onset and offset of the concentration change, and the duration of the stimulus period and the stimulus interval. As the three forms of concentration changes starts from the same initial concentration to the same end concentration, the amplitude of the concentration change alone may not determine the responses. The rate of concentration change became the obvious choice of parameters. The ability to respond to the rate of change is a prerequisite for “pulse slope detection”. The difficulty is to sufficiently accurately establish the rates of change during transient concentration changes provided by concentration pulses. To describe what information about the dynamics of a concentration pulse was transformed and processed in ON and OFF AL neurons, sensitivity to concentration pulses was characterized in terms of imp/s per % concentration change (Δ%) rather than rate of concentration change (%/s).

### Pulses in odor concentration

**Fig. 3:**
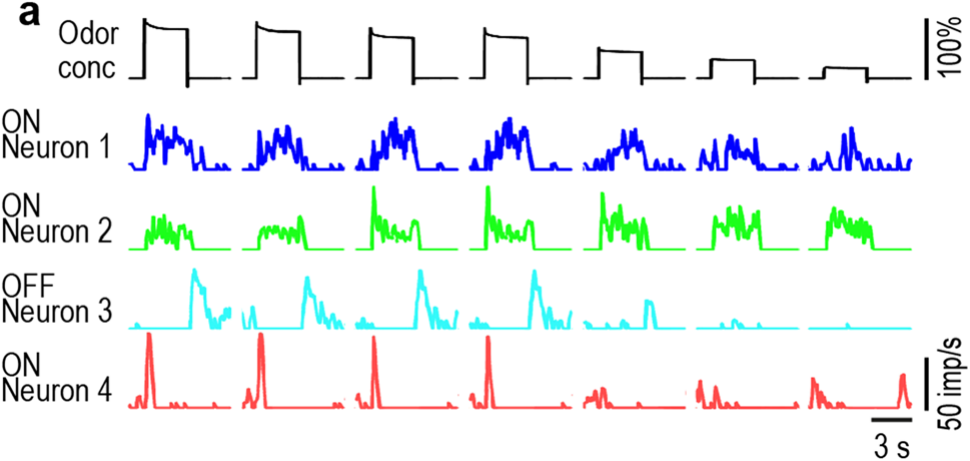
Tetrode recording of the activity of the same four AL neurons shown in Fig. 2 during a sequence of rectangular pulses of decreasing odor concentration. Top row: concentration time course of seven 3-s concentration pulses with 10-s pulse repetition intervals. The record is continuous; due to the restricted space the pulse intervals are not shown in full-length. Next rows: instantaneous discharge rates of neurons 1–4 presented as time histograms (bin width, 200 ms). ON-neuron 1 produces a rapid discharge increase to the concentration jumps, followed by a slow decline and a turn off after pulse termination. Response magnitude decreases with decreasing pulse amplitude. ON-neuron 2 generates a moderate discharge increase to the concentration jumps but also peak discharge rates, followed by a moderate, maintained activity and a brief after-discharge outlasting pulse duration. Response magnitude remains unchanged when pulse concentration decreases. The OFF-neuron 3 produces a phasic response at the concentration drops, followed by a decline in the discharge rate. Response magnitude decreases with decreasing pulse amplitude. The ON-neuron 4 is a highly phasic neuron producing a rapid discharge increase to the concentration jumps followed by a rapid decline to zero before the pulse end is reached. Responses to high pulse concentrations are strong, but they are weak to low pulse concentration, with an abrupt transition between the two activity levels

A sequence of 7 concentration pulses of lemon-oil odor with descending amplitudes from 97 to 18% was tested three times, but for descriptive purposes a single test sequence is shown in Fig. 3. ON-neuron 1 produced a large phasic response to strong concentration jumps, followed by an irregular discharge throughout the pulse. An after discharge outlasted the concentration drop and resumed to the initial ongoing activity. At low concentration jumps, the phasic component was absent and the discharge rate rose monotonically during the pulse duration. The response magnitude decreased with decreasing jump concentration. ON-neuron 2 had a phasic discharge at moderate concentration changes, followed by a sustained, irregular activity with brief peaks after pulse off. At strong and low concentration jumps, the phasic component was often reduced and preceded a plateau of stable activity. The response magnitude remained almost unchanged when jump concentration decreased. The odorless pulse intervals suppressed spontaneous activity. OFF-neuron 3 responded to concentration drops with a rapid phasic discharge, followed by a gradual return to the spontaneous activity. At low concentration drops the phasic discharge was reduced or even absent. The response magnitude decreased with decreased drop concentration. ON-neuron 4 produced a strong phasic discharge to large concentration jumps and a weak phasic discharge to small concentration jumps, with a sharp transition between large and small jumps. The activity ceased during the pulse periods and was absent throughout the pulse intervals. The four AL neurons generate both dynamic and static responses to the same concentration pulse.

**Fig. 4:**
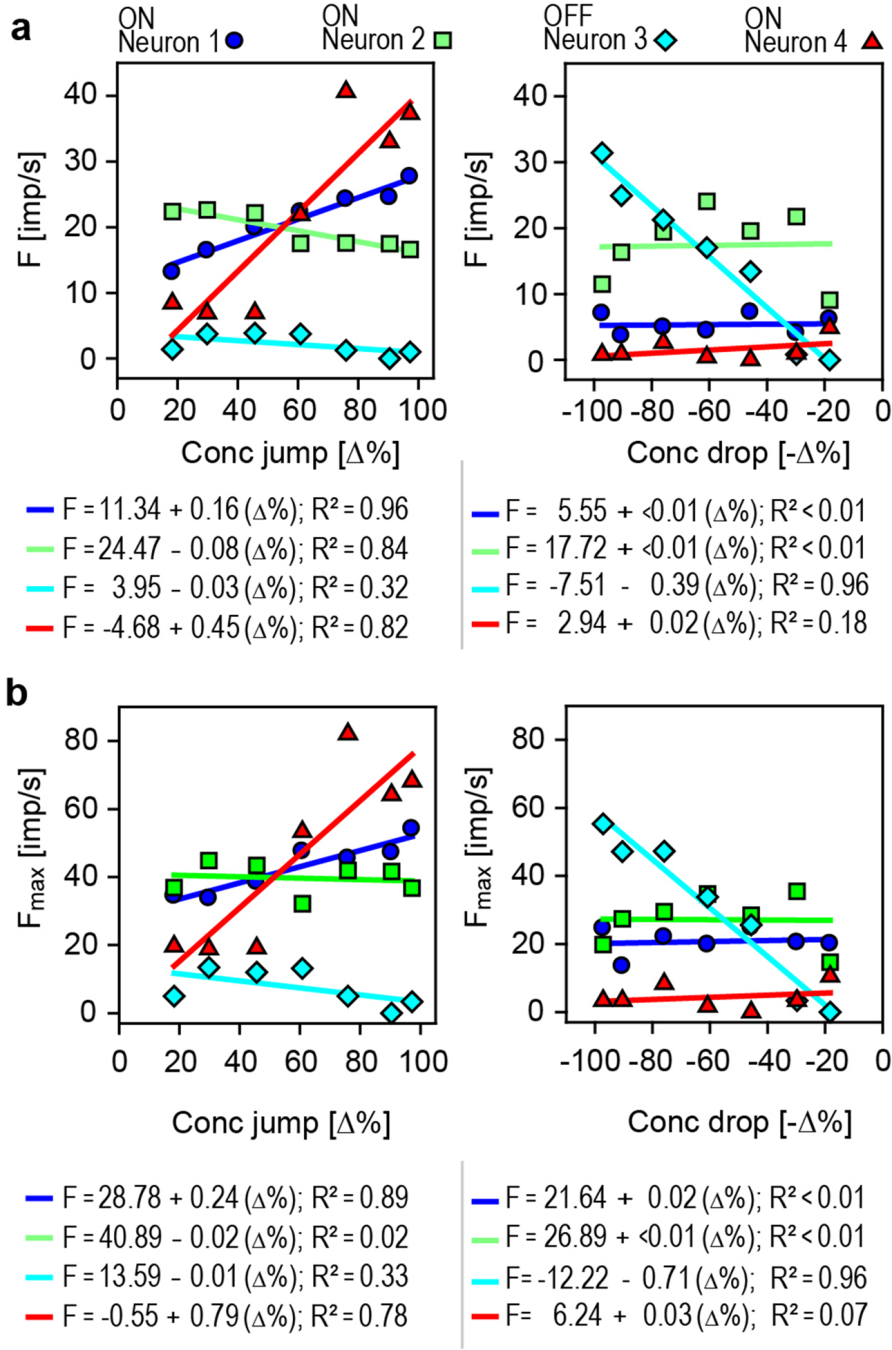
Response functions of the same four AL neurons shown in Figs. 2 and 3 obtained from a sequence of rectangular pulses of decreasing odor concentration delivered to antennal ORNs. **a** Left side: mean impulse frequencies plotted as linear functions of jump concentration (Δ%). The functions are positive and steep for ON-neurons 1 and 4, but they are negative and flat for ON-neuron 2 and OFF-neuron 3. The high *R*^2^ values for ON-neurons 1 and 4 indicate a strong dependence of impulse frequency on jump concentration, and the low *R*^2^ values for ON-neuron 2 and OFF-neuron 3 reveal a weak if any dependence. Impulse frequency of the ON-neurons 1 and 4 increases with increasing jump concentration, but in ON-neuron 2, impulse frequency slightly decreases with increasing jump concentration. The results from the regression equations are indicated under each diagram. Impulse frequency (*F*) determined by the number of impulses falling in the 3-s duration of the concentration pulse. Right side: mean impulse frequencies plotted as linear functions of drop concentration (− Δ%). The function for OFF-neuron 3 is positive and steep, but negative and flat for ON-neurons 1, 2 and 4. The high *R*^2^ for OFF-neuron 3 implies that the regression line is a perfect fit, and the low *R*^2^ for ON-neurons 1, 2 and 4 indicates no relationship. Impulse frequency of OFF-neuron 3 increases with increasing drop concentration. The results from the regression equations are indicated under each diagram. Impulse frequency (*F*) determined by the number of impulses falling in the 3-s period beginning with the concentration drop. **b** Left side: maximum impulse frequencies of the four neurons obtained during the 3-s pulse plotted as a linear function of jump concentration (Δ%). As expected from the mean-frequency plots, the maximum frequency of the two ON-neurons 1 and 4 increases with increasing jump concentration, but in contrast to the mean-frequency function, the maximum frequency of ON-neuron 2 is independent of jump concentration. Similar to the mean frequency, the maximum frequency of OFF-neuron 3 displays no dependence on jump concentration. Right side: maximum frequency values of the four neurons plotted as a linear function of drop concentration (− Δ%). As described for the mean frequencies, only the maximum frequency of OFF-neuron 3 increases with increasing drop concentration. The results from the regression equations are indicated above each diagram

The neurons’ sensitivity to transient upward and downward concentration changes was estimated by the linear regressions. Each regression was calculated for three consecutive series of odor pulses, and the response magnitude was determined by the cumulative impulse count during 3-s periods beginning with the onset of the jump or drop in concentration. The course of the regressions in the left diagram of Fig. 4a indicates that the impulse frequency of ON-neuron 1 increased with the size of the concentration jump (*R*^2^ = 0.96). The gain was 0.16 imp/s per % jump concentration, which means that an increase in 1 imp/s can be elicited by increasing the concentration jump by 6.2%. In ON-neuron 2, a linear function described the relationship between impulse frequency and jump concentration quite well (*R*^2^ = 0.84); the negative correlation indicates that the elevated activity decreased with increasing concentration jump. The gain was low, only − 0.08 imp/s per % jump concentration, meaning that the concentration jump must decrease by 12% to increase impulse frequency by 1 imp/s. OFF-neuron 3 was not affected by concentration jumps (*R*^2^ = 0.32). In ON-neuron 4, the linear regression fitted the relationship well (*R*^2^ = 0.82). The gain was 0.45 imp/s per % jump concentration, suggesting that jump concentration must be increased by 2.2% to raise the impulse frequency by 1 imp/s. The regressions in the right diagram of Fig. 4a show no dependence of the ON-neurons 1, 2 and 4 on drop concentration (*R*^2^ < 0.18), but OFF-neuron 3 increased impulse frequency with increasing drop concentration (*R*^2^ = 0.96). The slope of the regression indicates a gain value of 0.39 imp/s per concentration drop, requiring an increase in drop concentration of 2.5% for an increase of 1 imp/s. The relatively high activity of ON-neuron 2 reflects the sustained discharge, which outlasts the duration of the 3-s pulse periods.

The ON and OFF neurons exhibited peak discharge rates during the first second of the response to jumps and drops in concentration, respectively, in particular when the concentration change was large (Fig. 3). In Fig. 4b, peak frequency (bin width 0.2 s) of the four neurons was plotted as a function of the concentration changes, and the stimulus response functions were approximated by linear regressions. As peak frequency values were much higher than the cumulative impulse counts, the slopes of the two ON-neurons 1 and 4 became steeper by a factor 1.5 for concentration jumps (left graph), and the slope of the OFF-neuron 3 by a factor 2.5 for concentration drops (right graph). The *R*^2^ values indicate a similar good fit, suggesting that the scatter of the frequency values about the regressions remained the same. This observation raised the question of whether peak frequency values would reflect transient concentration changes with a higher accuracy than mean frequencies. The gain of the peak response of ON-neuron 1 was 0.24 imp/s per % concentration jump, and for ON-neuron 4, 0.79 imp/s per % concentration jump. As indicated by the reciprocal of the slope values, the concentration jump must be increased by 4.2% to raise the impulse frequency of ON neuron 1 by 1 imp/s; ON-neuron 4 was more sensitive, requiring an increase in concentration jump of only 1.3% (left graph). For the peak response of OFF-neuron 3, gain was 0.71 imp/s per % concentration drop, needing an increase in drop concentration of 1.4% for an increase of 1 imp/s (right graph).

**Fig. 5:**
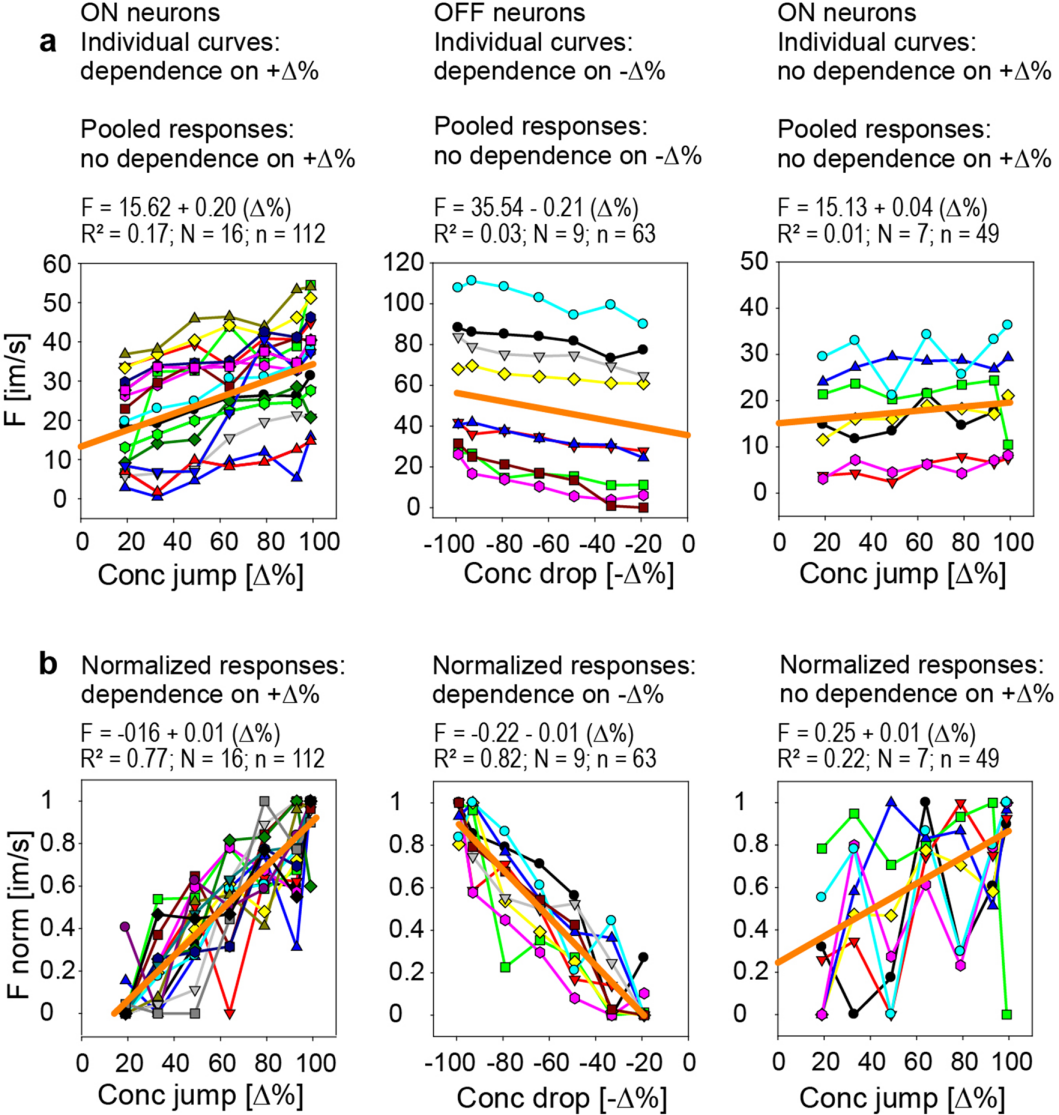
Response functions of 32 single ON and OFF neurons for a sequence of seven, triple-tested rectangular pulses of decreasing odor concentration delivered to antennal ORNs. **a** Left side: 16 ON-neurons exhibit an increase in impulse frequency with increasing jump concentration. Middle: 9 OFF-neurons generate an increase in impulse frequency with increasing drop concentration. Right side: 7 ON-neurons produce an excitatory response to concentration jumps that is invariant to jump concentration. The stimulus–response relationship shown for each neuron by a line graph was validated by fitting a linear regression and quoting *R*^2^ statistics, as outlined in the text. Pooling the responses across each neuron group and approximating the course of the cumulated responses by single linear regressions (orange lines) yields low *R*^2^ values, which indicate no correlation between the average ON-neuron’s response on jump concentration and the average OFF-neuron’s response on drop concentration. **b** Responses of the each neuron are normalized to its maximum frequency value and pooled across each neuron group for regression analysis to estimate the relationship between the cumulative normalized responses and pulse concentration. For the 16 ON-neurons showing a dependence on jump concentration (left side), the average gain value of normalized responses is 0.01 imp/s per % increase in jump concentration; for the 9 OFF-neurons depending on drop concentration (middle), the average gain value of normalized responses is 0.01 imp/s per % increase in concentration drop. *R*^2^ is 0.77 for the ON neurons, 0.82 for the OFF neurons. For the 7 ON-neurons showing no dependence on jump concentration (right side), *R*^2^ is 0.22. Impulse frequency (*F*) determined by the number of impulses falling in the 3 s of the pulse duration or the 3-s period beginning with the concentration drop. The results from the regression equations are indicated above each diagram

As illustrated in Figs. 2 and 3, individual AL neurons responded quite differently to the same lemon-odor concentration pulse. They can be sub-divided into three groups according to the effect of the concentration amplitude. The first group consists of ON neurons whose responses to concentration jumps increased with jump concentration (Fig. 5a, left graph), the second group contained OFF neurons whose responses to concentration drops increased with drop concentration (Fig. 5a, middle graph), and the third group encompassed ON neuron that exhibited concentration-invariant responses to concentration jumps (Fig. 5a, right graph). The line graphs in Fig. 5a differ in their position or height on the frequency axis (*y*-intercept) indicating broad ranges of response minima and maxima elicited with low and high concentration pulses, respectively. However, the overall rate at which the response frequency of a neuron increased with further change in concentration was similar within a group. Linear regressions were used to quantify for each neuron the individual relationship between impulse frequency and pulse concentration. For the ON neurons showing a dependence on jump concentration (Fig. 5a, left graph), the mean gain value was 0.20 imp/s per % increase in jump concentration, and for the OFF neurons with a dependence on drop concentration (Fig. 5a, middle graph), the mean gain value was  0.21 imp/s per % increase in drop concentration. The reciprocal of the average slope of the ON neurons reveals that the concentration jump must be increased by 5.0% to raise the impulse frequency by 1 imp/s; the average slope of the OFF neurons suggests a required increase in the concentration drop of 4.7% to raise impulse frequency by 1 imp/s. The basic statistics are shown in Table 1.

**Table 1:** Summary of data used to determine gain of the ON and OFF neurons for concentration pulses

Type of neuron	ON neuron	OFF neuron	ON neuron
	Dependence on + Δ%	Dependence on − Δ%	No dependence on + Δ%
Range of concentration jumps and drops (Δ%)	18–97	− 97 to − 18	18–97
Neurons used for stimulus–response relations	16	9	7
Spontaneous activity (imp/s)	14.40 ± 7.20	42.06 ± 5.21	6.34 ± 1.96
*Single linear regressions*			
Number of linear regressions	16	9	7
Number of points per linear regression	7	7	7
Mean *y*0-intercept of regressions (imp/s)	15.62 ± 12.66	35.54 ± 35.09	15.13 ± 10.33
*y*0-intercept values range	− 4.68 to 32.88	− 7.50 to 87.99	2.20 to 26.75
Mean gain (mean *a* value, imp/s per Δ%)	0.20 ± 0.09	− 0.21 ± 0.07	0.03 ± 0.04
Gain values range (imp/s per Δ%)	0.10 to 0.44	− 0.10 to − 0.38	− 0.06 to 0.08
Mean coefficient of determination (*R*^2^)	0.79 ± 0.12	0.87 ± 0.06	0.35 ± 0.22
*R*^2^ values range	0.59 to 0.96	0.78 to 0.96	0.10 to 0.47
*Linear regression from pooled responses*			
Number of linear regressions	1	1	1
Number of points per linear regression	112	63	49
*y*0-intercept of regression (imp/s)	15.62	35.54	15.13
Gain (*a* value, imp/s per Δ%)	0.20	− 0.21	0.04
Coefficient of determination (*R*^2^)	0.17	0.03	0.01
*Single linear regressions from normalized responses*			
Number of linear regressions	16	9	7
Number of points per linear regression	7	7	7
Mean *y*0-intercept of regressions (imp/s)	− 0.14 ± 0.12	− 0.21 ± 0.10	0.24 ± 0.36
*y*0-intercept values range	− 0.37 to 0.08	− 0.34 to − 0.05	− 0.03 to 1.00
Mean gain (mean *a* value, imp/s per Δ%)	0.01 ± < 0.01	− 0.01 ± 0.01	< 0.01 ± < 0.01
Gain values range (imp/s per Δ%)	< 0.01 to 0.01	− < 0.01 to − 0.10	− < 0.01 to < 0.01
Mean coefficient of determination (*R*^2^)	0.79 ± 0.12	0.87 ± 0.06	0.37 ± 0.22
*R*^2^ values range	0.61 to 0.96	0.77 to 0.95	0.10 to 0.40
*Linear regression from pooled normalized responses*			
Number of linear regressions	1	1	1
Number of points per linear regression	112	63	49
*y*0-intercept of regression (imp/s)	− 0.16	− 0.22	0.25
Gain (*a* value, imp/s per Δ%)	0.01	0.01	0.01
Coefficient of determination (*R*^2^)	0.77	0.82	0.22

Mean values include ± SD

While the responses of individual neurons within each group are variable in absolute terms, they nonetheless maintain a relatively constant relationship with one another during series of concentration pulses. This raises the question how the information of concentration pulses transmitted and passed by individual AL neurons is combined by target neurons to provide the maximal possible transfer of transient concentration changes to the brain. A simple combinatorial process would be summing or averaging the responses of individual AL neurons, in which target neurons in no way differentiate the inputs of the different AL neurons. If the neurons in the group have similar response characteristics, this simple summing of responses may greatly improve the definition of the stimulus above what possible by single neurons. To verify this, the regression analysis was performed by pooling the responses across the ON neurons and OFF neurons. However, approximating the course of the cumulative points by a single linear regression (Fig. 5a, orange lines; Table 1) eliminates any dependence of the ON-neurons’ responses on concentration jumps and of the OFF-neurons’ responses on concentration drops.

Another combinatorial process involves adjusting the responses of individual AL neurons to a common scale, without distorting differences in the ranges of the response magnitudes. Normalizing individual responses to the maximum frequency values of each neuron and then pooling the normalized responses across the neurons of each group yielded fitted regression lines with diagonal slopes of approximately 45° (Fig. 5b, orange lines; Table 1). The functions, however, did not go through the origin. Nevertheless, the relative impulse frequency and pulse concentration had a proportional relationship. The cumulative, relative impulse frequency provided a group estimate for the neurons’ response gain. For the ON neuron, the mean indicated that the normalized gain value was 0.01 imp/s per % concentration jump, for the OFF neuron the normalized gain value was − 0.01 imp/s per % concentration drop. As indicated by the reciprocal of gain values, an increase in the concentration jump by 1% results in an increase in the ON neurons’ relative activity of 1% of the full frequency scale, and an increase in the concentration drop by 1% produces an increase in the OFF neurons’ activity of 1% of the full frequency scale.

The analysis demonstrates that, for a given group of ON or OFF neurons, the differentiation of the pulse concentration is realistic if the measure of the group response was the actual discharge rate of individual neurons and the brain differentiated the inputs of the different fibers. Simple summing or averaging the responses of individual neurons, however, lost the resolution of pulse concentration. Even though the neuron samples were relative uniform in the gain for concentration pulses, they differ in their response threshold. This yields a high variability of the cumulative response, making it impossible to detect any relationship on concentration pulses. Optimally scaling the contribution of each neuron to the measure of the group response improved the pulse-concentration resolution.

The ON and OFF neuron responses not only represent pulse concentration but reflect, implicitly at least, information about the rate at which concentration changes. The common view, however, is that impulse frequency is the response to pulse concentration alone: almost all studies dealing with this issue showed that impulse frequency takes on different values with different values of pulse concentration. As the slope of the concentration pulse defines a change in concentration over time, the rate of change may also determine the neuron’s impulse frequency (beyond the concentration amplitude). The result will be a double dependence of impulse frequency on pulse concentration and the rate of changes required to reach the level of pulse concentration. Describing what information on the dynamics of concentration changes is contained in the activity of AL neurons requires changing the two parameters of the odor stimulus, namely, concentration and its rate of change, independently of each other. We did this by oscillating the changes in odor concentration.

### Oscillations in odor concentration

**Fig. 6:**
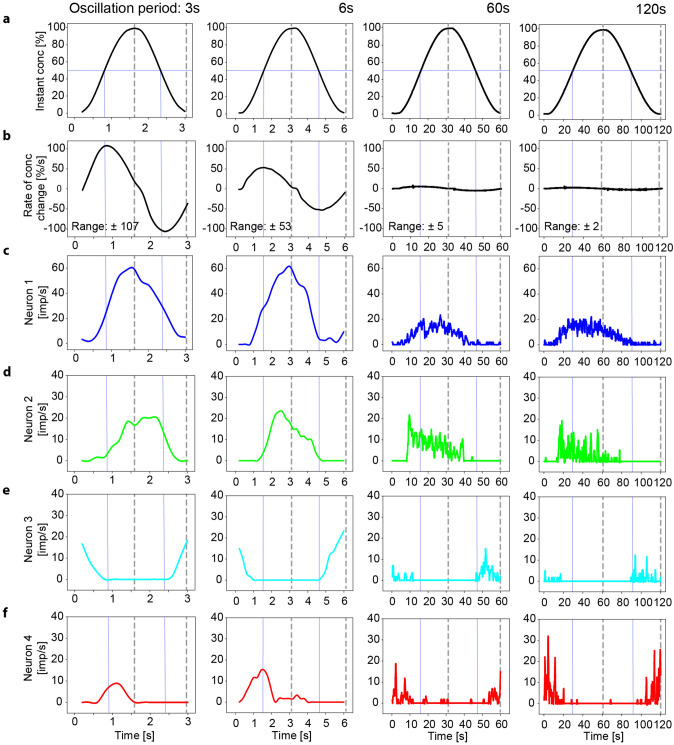
Simultaneously recorded responses of the same 4 AL neurons shown in Figs. 2, 3 and 4 to oscillating concentration changes. **a** Time course of odor concentration oscillating at constant amplitude (0–100%) over four different period durations. Different time scales are used to demonstrate complete oscillation periods. **b** Time course of the rate of concentration change. The maxima of the oscillating rate-of-change are one quarter of the period duration in advance of the maxima of the oscillating concentrations. **c–f** Time course of the neurons’ impulse frequencies. **c** Impulse frequency of ON-neuron 1 is high during brief oscillation periods (3 and 6 s) and low during long oscillation periods (60 and 120 s); frequency maxima are in phase with concentration maxima during brief periods (3 and 6 s), but ahead of the concentration maxima and behind the maxima of the rate of change during long oscillation periods (60 and 120 s). **d** Frequency maxima of ON-neuron 2 decrease with increase period duration, showing at the same time an increasing phase advance to the concentration maxima. During the longer periods (60 and 120 s), the discharge peaks at the beginning of the up cycle of the concentration change. **e** OFF-neuron 3 produces a peak discharge at the beginning of the down cycle of the concentration change, and the frequency maxima decrease with increasing duration of the oscillation period. **f** Frequency maxima of ON-neuron 4 are ahead of the concentration maxima and slightly behind the maxima of the rate of change. The phase advance and the frequency maxima increase with the period duration. Dotted vertical lines indicate the phase shift between time courses of odor concentration, impulse frequency and rate of concentration change. The same instantaneous concentration within a given oscillation period (horizontal line in **a**) can be accompanied in each neuron by two different values of impulse frequency (vertical lines)

Figure 6 illustrates the responses of the same four neurons shown in Figs. 3 and 4 during constant-amplitude oscillations with periods of 3, 6, 60 and 120 s. The upper two panels show the time course of the oscillating concentration and the corresponding oscillating rate at which concentration changes (Fig. 6a, b). The maxima of the oscillating rate of change are one quarter of the full period in advance of the maxima of the oscillating concentration. The lower four panels show the rate of discharge of the 4 neurons using a bin width of 0.2 s, which results in 30 bins for the relatively brief 6-s oscillation period (Fig. 6c–f). ON-neuron 1 was continuously active through the full cycles of the oscillations, ON-neurons 2 and 4 were active through the up cycles and fell silent through the down cycles, and OFF-neuron 3 was active through the down cycles but not through the up cycles. The maximum frequency of each neuron decreased with the duration of the oscillation period.

As shown in Fig. 6c, the impulse-frequency profile of ON-neuron 1 was smooth during brief oscillation periods (3 and 6 s), and frequency rose up to 60 imp/s when the concentration increased and declined to almost zero when the concentration decreased. During long periods (60 and 120 s), the profile was irregular and the frequency maxima were about one-third compared to brief periods. The mean frequency during the up cycles of the 60-s and 120-s periods was similar: 6.49 ± 6.19 imp/s (*n* = 300) and 6.89 ± 6.23 imp/s (*n* = 600), respectively. The irregularity in the spike train was quantified using the coefficient of variation (CV, defined as the ratio between SD and mean frequency). The variation values were 0.95 for the 60-s period and 1.16 for the 120-s period. During brief oscillation periods (3 and 6 s), the frequency maxima were in phase with the concentration maxima, during long oscillation periods (60 and 120 s), the frequency maxima were in advance of them. The impulse-frequency profile of ON-neuron 2 (Fig. 6d) was also smooth during brief oscillation periods (3 and 6 s). The frequency maxima were in phase with the concentration maximum during the 3-s period but ahead of it during the 6-s period. During the longer 60- and 120-s periods, frequency rose faster than concentration and the frequency maxima for the two periods were quite similar, namely, 21 imp/s for the 60-s period and 18 imp/s for the 120-s period. The mean frequency values during the two up cycles differed (*p* < 0.01), they were 6.31 ± 5.03 imp/s (*n* = 160) for the 60-s period and 3.97 ± 4.14 imp/s (*n* = 270) for the 120-s period. The spike train irregularity (CV) for the up cycle of the 60-s period was 0.79 and 1.04 for the up cycle of the 120-s period. During the longer 60- and 120-s periods, the frequency maxima were ahead of the concentration maxima. OFF-neuron 3 (Fig. 6e) produced a continuous and smooth discharge during periods of 3 and 6 s just as the oscillating concentration passed the minimum values and then increased. During the longer periods of 60 and 120 s, a phasic peak discharge occurred right at the minimum concentration. In ON-neuron 4 (Fig. 6f), the discharge increased smoothly to a maximum, which was in advance of the maximum values of the concentration during both the 3- and 6-s periods. At longer periods of 60 and 120 s, the phasic peak discharges was also ahead of the maximum values of the instantaneous concentration but occurred just at the maximum values of the rate of change. Note that the amplitude of maximum frequency values increased with the period duration.

**Fig. 7:**
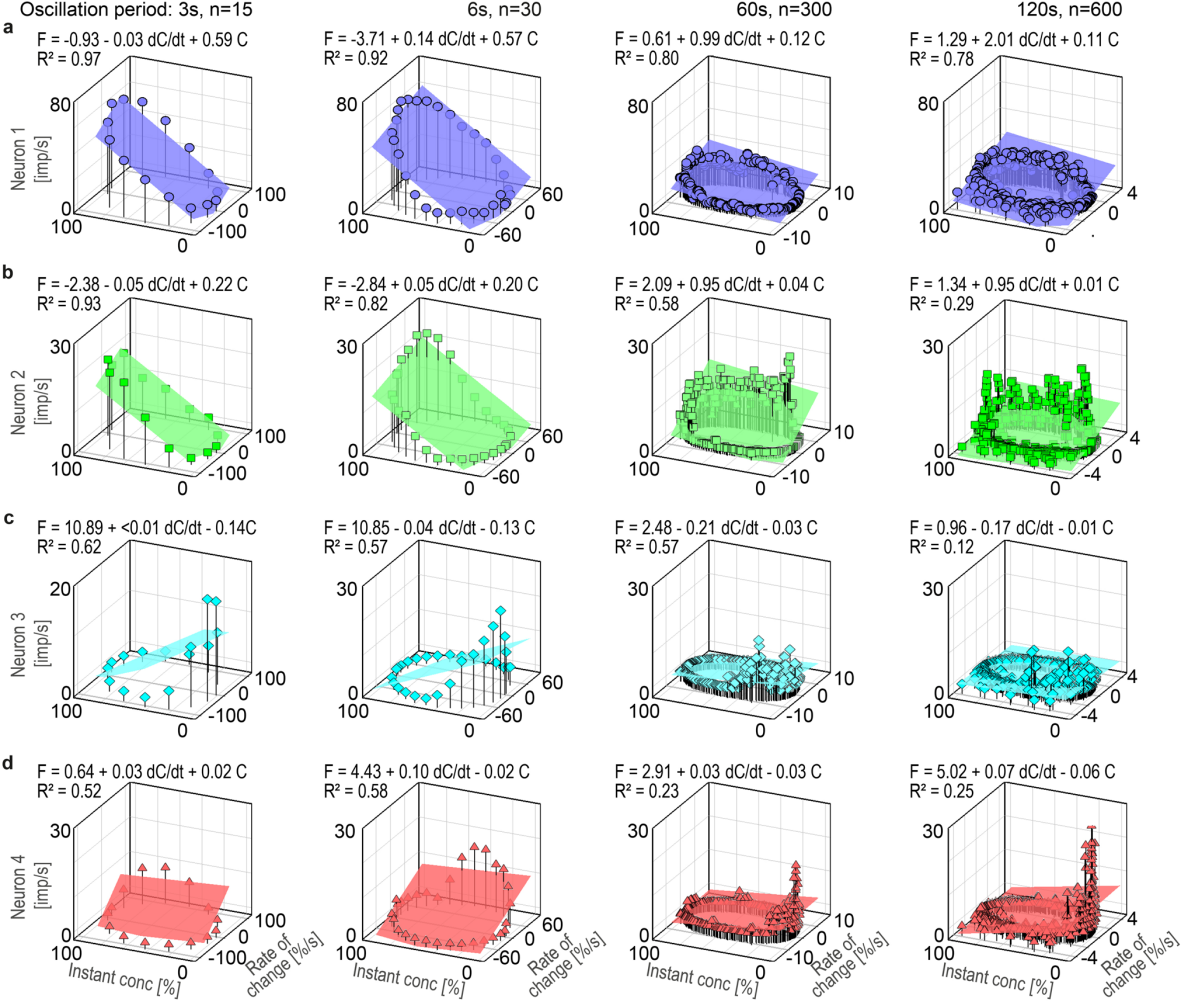
Impulse frequency of the same 4 AL neurons in Fig. 5 during 4 periods of oscillating odor concentration plotted as a function of the instantaneous concentration and the rate of change. Multiple regressions that utilize three-dimensional planes were calculated to determine the gain of responses for the instantaneous odor concentration and the rate of concentration change. **a** Impulse frequency values of ON-neuron 1 closely fit the slopes of the regression planes (*R*^2^ ≥ 0.78), indicating a double dependence on the instantaneous concentration and its rate of change. Increasing the duration of the oscillation period leads to a decrease in the gain for the instantaneous concentration and an increase in the gain for the rate of change. **b** ON-neuron 2 shows a similar double dependence like ON-neuron 1. Impulse frequency and gain values are lower, the values of *R*^2^ (≥ 0.82) for periods of 3 and 6 s represent the relatively small distance between the data values and the fitted values. For 60 and 120 s periods, impulse frequency values are not close to the regression planes, hence the relationship is good (*R*^2^ = 0.58) and low (*R*^2^ = 0.29), respectively. The *p* values (*p* < 0.001) for each independent variable, the instantaneous concentration and its rate of change indicate statistically significant correlation with impulse frequency. **c** OFF-neuron 3 produces short-duration responses at the falling concentration cycles, suggesting limited usefulness of regression planes for describing the relationship between the impulse frequency and the two parameters of the oscillations in concentration over the entire range of rates. *R*^2^ values are good for periods of 3, 6 and 60 s (*R*^2^ ≥ 0.57), and low (*R*^2^ = 0.12) for the long period of 120 s. The *p* values (*p* < 0.01) indicate statistically significant correlation of the impulse frequency and the instantaneous concentration for brief periods of 3 and 6 s, and strong evidence for a double dependence on the instantaneous concentration and its rate of change for long periods of 60 and 120 s. Note that the slopes for the instantaneous concentration are orientated in the opposite direction to the ON neurons. **d** Impulse frequency of ON-neuron 4 displays a pronounced excitatory response of brief duration to the rising concentration cycles, pointing to limited usefulness of regression planes for describing the relationship between the impulse frequency and the two parameters of the oscillations in concentration for the entire range of rates tested. *R*^2^ values are medium for brief periods (*R*^2^ ≥ 0.52) and low for long periods (*R*^2^ ≥ 0.23). The *p* values (*p* < 0.001) indicate a statistically significant correlation between the impulse frequency and the rate of concentration for periods of 3 and 6 s, and between the impulse frequency and the instantaneous concentration for periods of 60 and 120 s

The phase relationship between the oscillations in impulse frequency and the oscillations in the instantaneous concentration has the tendency to vary with the duration of the oscillation period. With increasing oscillation period the frequency maxima tended to lead the concentration maxima and to lag behind the rate-of-change maxima. In Fig. 7, impulse frequencies of the ON and OFF neurons were plotted as a function of the instantaneous concentration and its rate of change. The frequency curves approached closed circles similar to Lissajous figures, in which two oscillating parameters are plotted, one as a function of the other. The shape of these figures is determined by the ratio of the two oscillating parameters, or more specifically, the ratio of their amplitudes and their phase differences. Multiple regressions were calculated to determine the simultaneous effect of the instantaneous concentration (*b* slope) and the rate of change (*a* slope) on the response frequency during different oscillation periods.

In ON-neuron 1, the coefficients of determination (*R*^2^ ≥ 0.78) indicate a strong linear relationship between the impulse frequency, the instantaneous concentration and the rate of concentration change for each of the 4 oscillation periods (Fig. 7a). According to the regression slopes, the gain for the instantaneous concentration decreased with the duration of the oscillation period, from 0.59 imp/s per % for the 3-s period to 0.11 imp/s per % for the 120-s period. Conversely, the gain for the rate of change increased with the period duration, from 0.03 imp/s per %/s for the 3-s period to 2.01 imp/s per %/s for the 120-s period. Although the two parameters, instantaneous concentration and its rate of change, cannot be set in direct relationship to each other, their effects on impulse frequency can be by determining that increment of each parameter which results in the same increment in impulse frequency. The measurements showed that an increase of 1 imp/s can be elicited during an oscillation period of 3 s either by a 1.7% increase in the instantaneous concentration (provided the rate of change is constant) or by a rate of change of 33%/s. During a 120-s oscillation period, it takes an increase of 9% in the instantaneous concentration to increase impulse frequency by 1 imp/s, or a rate of change of 0.49%/s. Thus, during brief oscillation periods, impulse frequency could be influenced more by changing the instantaneous concentration by 1% than by changing the rate of concentration change by 1%/s. Conversely, however, during long oscillation periods, impulse frequency could be influenced more by changing the rate of concentration change 1%/s than by changing the instantaneous concentration by one additional degree.

In ON-neuron 2, the coefficients of determination (*R*^2^ ≥ 0.82) for the brief oscillation periods of 3 and 6 s indicate a strong linear relationship between the impulse frequency and the two components of the odor stimulus. For the 60-s period, the multiple regression provides a good fit to the frequency values (*R*^2^ = 0.58), but for the 120-s period (*R*^2^ = 0.29), a quadratic relationship may fit the data better than a linear one (Fig. 7b). Checking the regression analysis, however, revealed a significant dependence of the impulse frequency on both the instantaneous concentration and the rate of change (*p* < 0.01). This was accompanied by a large variance in the *y*-intercept (*p* < 0.02), meaning that the height of the plane is not interpretable. The large variance is explained by the discontinuous activity: the rapid increase in the discharge rate, which occurred directly at the increase in concentration, was followed by remarkably irregular spike trains, declining slowly to zero (Fig. 6c). As indicated by the regression slopes, the gain values for the two brief periods (3 and 6 s) were the same, as was also the case for the two long periods (60 and 120 s). During the brief 3-s period, the gain for the instantaneous concentration was 0.22 imp/s per % and the gain for the rate of change was 0.05 imp/s per %/s. An increase of 5% in the instantaneous concentration has the same effect on impulse frequency as a 20%/s increase in the rate of change. Both result in a 1 imp/s increase in impulse frequency. During the long 60-s period, the gain for the instantaneous concentration was 0.04 imp/s per % and the gain for the rate of change was 0.95 imp/s per %/s. An increase of 1 imp/s can be elicited either by a 25% increase in the instantaneous concentration or by a 1.05%/s rate of change. During brief periods, impulse frequency can be altered more by changing the instantaneous concentration by 1% than by changing the rate of concentration change by 1%/s. Conversely, during long periods, impulse frequency can be altered more by changing the rate of concentration change by 1%/s than by changing the instantaneous concentration by one additional degree.

In OFF-neuron 3, the coefficients of determination (*R*^2^ ≥ 0.57) for oscillation periods of 3, 6 and 60 s indicate that the regression planes represent a good approximation of the relationship between the impulse frequency, the instantaneous concentration and the rate of concentration change (Fig. 7c). The statistical evaluation of the regression analysis, however, revealed that during brief periods (3 and 6 s) the impulse frequency was not influenced by the rate of change (*p* < 0.48), only by the instantaneous concentration (*p* < 0.01). During the 3-s period, the gain for the instantaneous concentration was − 0.14 imp/s per %, meaning that a 7% decrease in the instantaneous concentration is required to increase the impulse frequency by 1 imp/s. During the long periods of 60 and 120 s, the horizontal axes of the regression planes and the low coefficient of determination (*R*^2^ ≥ 0.12) suggest a poor if any dependence of impulse frequency on the two parameters of the odor stimulus. They were, however, well-correlated with impulse frequency (*p* < 0.01). During the long 60-s period, the gain for the instantaneous concentration was − 0.03 imp/s per % and the gain for the rate of change was − 0.21 imp/s per %/s. An increase of 1 imp/s can be elicited either by a 33% decrease in the instantaneous concentration or by a rate of change of − 4.7%/s. Impulse frequency can be influenced more by changing the rate of concentration change by 1%/s than by changing the instantaneous concentration by one additional degree.

In ON-neuron 4, the coefficients of determination ranged between a good fit (*R*^2^ ≥ 0.52) for brief periods (3 and 6 s) and a poor fit (*R*^2^ ≥ 0.23) for the long periods (60 and 120 s), but the *p* values (< 0.01) for brief periods indicates a dependence on the rate of change, and for long periods on the instantaneous concentration. During brief periods, the gain for the rate of change was 0.02 imp/s, indicating that an increase of 1 imp/s can be elicited by a 50% increase in the rate of change. During long periods, the gain for the instantaneous concentration was 0.03 imp/s, suggesting that a 1 imp/s increase can be elicited by a 33% increase in the instantaneous concentration. During brief oscillation periods, the gain of response can be altered by changing the rate of concentration change, but during long oscillation periods by changing the instantaneous concentration.

Figure 7a, b shows that the gain for the rate of change of ON-neurons 1 and 2 tended to be low during brief oscillation periods and increased during long oscillation periods. Conversely, the gain for the instantaneous concentration tended to be high during brief periods and decreased during long periods. Thus, during long oscillation periods, ON neurons 1 and 2 increased the gain for the rate of change at the expense of the gain for the instantaneous concentration. This trade-off indicates that the neurons do not simply transform fluctuations in odor concentration, but balance—from instant to instant—their gain according to stimulus conditions. OFF-neuron 3 exhibits a similar gain control during long periods, but during brief periods the gain for the instantaneous concentration is not traded for the rate of change. Thus, the gain for the instantaneous concentration is not influenced by variations in the duration of the oscillation period. In ON-neuron 4, no gain control and resulting trade-off was found. During brief oscillation periods, the gain for the rate of change is not affected by the instantaneous concentration; and during long periods, the gain for the instantaneous concentration is not affected by the rate of change.

**Fig. 8:**
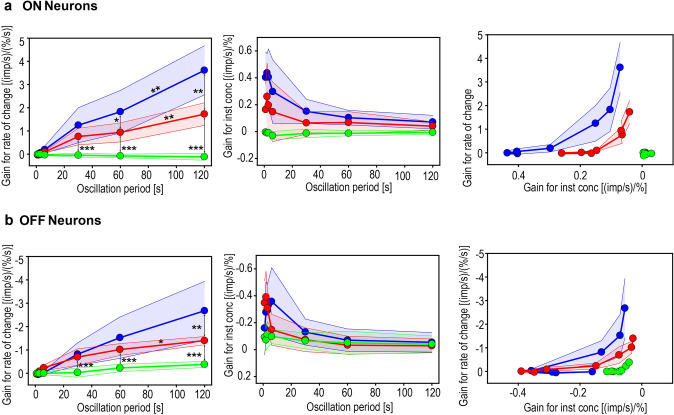
Effect of the duration of the oscillation period on the gain of **a** 18 ON neurons and **b** 18 OFF neurons. Gain values are obtained for each period from regression planes by calculating impulse frequency as a function of instantaneous concentration and its rate of change. Both types of neurons can be subdivided into 3 groups, each with 6 neurons, according to the dependence of the gain for the rate of concentration change on the period duration: strong dependence (blue), moderate dependence (red), and no dependence (green). Mean values and standard error of the means obtained for each group are indicated. Left side: dependence of the mean gain for the rate of change on the duration of the oscillation periods. Middle: dependence of the mean gain for the instantaneous concentration on the duration of the oscillation periods. Right side: for each period, the mean gain for the rate of change is plotted as a function of the mean gain for the instantaneous concentration. The negative values for gain reflect the downward direction of the concentration change, yielding an increase in impulse frequency of the OFF neurons. Differences in mean gain values between adjacent neuron groups at the same period or adjacent oscillation periods of the same neuron group were assessed by ANOVA, *p < 0.05; **p < 0.01; ***p < 0.001

The increase in the period duration from 1 to 120 s results in a decrease in the rate of concentration change by a factor of roughly one hundred. Gain control can, therefore, be interpreted as being an adaptation to variations in the range of concentration rates due to variations in the period duration. This can be illustrated by plotting the gain values obtained from the regression planes for each ON and OFF neuron against the oscillation period (Fig. 8). The resulting gain functions of individual neurons differ with respect to several parameters. These include the rate at which both gain values change with increases in period duration (gain slope) and the size of the maximum gain, with due consideration of sign. In general, the gain for the rate of change has steeper functions and higher gain maxima than the gain for the instantaneous concentration, sign ignored. Furthermore, the gain values show a high variability. In spite of this variability, simple inspection of Fig. 8 points to general trends in some variable in relation to another. To help compare these trends, the relationships between the gain functions of the ON and OFF neurons and the period duration were categorized as strong (group 3), moderate (group 2), or non-existent (group 1), shown by the colored gain functions (Fig. 8). Grouping was done manually with a sense of proportion, and the subdivision between strong and moderate gain values for the rate of change corresponds with a value set at 2 imp/s per %/s for the 120-s period. If a neuron’s gain maximum for the rate of change was closely above or below this value, the gain slope was used to verify grouping. These criteria seem admittedly somewhat arbitrary, but nevertheless cover all neurons of the total recorded population, reveal general trends in the data, differences in the groups and overlap.

Across the entire range of oscillation periods, the average gain values of group-1 ON and OFF neurons are lower than the average gain values of groups 2 and 3 ON and OFF neurons (*p* < 0.01). During the 120-s period, group 2 ON and OFF neurons have on average a lower gain than group-3 ON and OFF neurons (*p* < 0.01), and the gain of group-2 ON neurons is on average lower during the 60-s versus 120-s period (*p* < 0.01). In addition, during the 60-s period, group 2 ON neurons are on average lower than group-3 ON neurons (*p* < 0.05). The diagrams indicate that the gain for rate of change and the gain for instantaneous concentration are inversely related to the period duration. Neurons with high gain values for the rate of change during long periods consistently show high gain values for the instantaneous concentration during brief periods. Thus, the ON and OFF neurons trade the gain for the rate of change at the expense of the gain for the instantaneous concentration (Fig. 8a, b, right graphs).

The mean gain values of group-3 ON neurons for the instantaneous concentration decreased from 0.40 ± 0.20 imp/s per % for the 3-s period to 0.07 ± 0.05 imp/s per % for the 120-s period (*p* < 0.001), and the mean gain values of group-3 OFF neurons decreased from − 0.16 ± 0.19 imp/s per % to − 0.05 ±  < 0.01 imp/s per %, respectively (*p* < 0.1), sign ignored. The mean gain values of group-3 ON neurons for the rate of change increased from 0.05 ± 0.11 imp/s per %/s for the 3-s period to 3.61 ± 1.05 imp/s per %/s for the 120-s period (*p* < 0.001), and their mean gain values increased from 0.01 ±  < 0.01 imp/s per %/s to − 2.67 ± 1.25 imp/s per %/s, respectively (*p* < 0.001), sign ignored. The gain for the rate of change is increased more than one hundred times in the presence of low rates of change during long oscillation periods, and the gain for the instantaneous concentration is increased about ten times in the presence of fast rates of change during brief oscillation periods. These effects occur at a speed equal to the fluctuating changes in odor concentration, indicating a substantial contribution to moment-to-moment odor processing.

## Discussion

A salient feature of the responses of the ON and OFF neurons to the instantaneous concentration of lemon-oil odor is the strong dependence on the rate with which concentration changes. Moreover, the ON and OFF responses do not differ just by their sign. The impulse-frequency values of the ON neurons span different ranges than the OFF neurons, which means that OFF neurons can respond with higher frequencies to concentration drops than ON ORN to equivalent concentration jumps, but ON neurons can respond with higher frequencies during the up cycles of concentration oscillations than OFF ORN to equivalent down cycles. Nonetheless, as the ON and OFF ORNs occur together in the same trichoid sensillum, both ORNs share the same receptive field and would receive the same concentration change. Thus, the physical parameter rate-of-change operating on the ON ORNs must be equal to that operating on the OFF ORNs. This means that the antagonistic response of the ON and OFF ORNs as well as the differences in the responsiveness within an antagonist cannot be attributed to random or systematic differences in the physical access of stimulus molecules to the receptor neuron membrane. Accordingly, not only the ON and OFF responses of ORNs but also those of the ON and OFF AL neurons must be evoked by the same rate of concentration change.

The ON and OFF neurons were identified by their excitatory responses to upward and downward concentration steps of lemon-oil odor, respectively. Since concentration increments and decrements do not occur physically at the same time in the same place, the polarity of the responses of the neurons to changes in odor concentration resembles complementary pairs of electronic amplifiers. Using a “push–pull” arrangement, one neuron acts during the positive concentration change and the other one during the negative change. Yet not only an increase, but also a decrease in impulse frequency can serve to convey information. However, the lower the impulse frequency become, the longer it takes to do so. If the concentration fluctuations are becoming faster, so that the direction of change can vary during the period needed for an ON neuron to transmit the extent and the rate of a decrease in concentration, the ON neuron cannot keep up. Its response will be interrupted and, therefore, less accurate. However, the OFF neuron will be able to keep up, because during decreasing odor concentration, its frequency is high. Since impulse frequency of one of the two neurons is always high during increasing and decreasing concentration, information can be supplied during both directions of change by one or the other neuron.

Odor-induced activity patterns are the result of both chemical and physical parameters of the odor stimulus. The experiments described here were focused on two mutually independent physical parameters, the odor concentration and its rate of change. Odor identity was not considered; response inhibition was not included in this study. Note that the designation of ON and OFF neurons refers to their antagonistic responses to changes in the concentration of the lemon-oil odor but should not result in a bias in specificity. A study of specificity should include a test for bimodal responses: intracellular recording and staining techniques have demonstrated that information about pulses of the lemon-oil odor, odor temperature and slow antennal displacements is integrated by AL neurons (Zeiner and Tichy 1998, 2000). This indicates that pulse-like concentration changes are embedded in bimodal events, which are encoded by bimodal AL neurons during combined stimulation. Therefore, it cannot be excluded that some ON and OFF neurons responsive to pulse concentration are involved in the integration of simultaneously occurring mechanical, temperature and olfactory stimuli, and other ON and OFF neurons not responsive to pulse concentration participate in the spatial glomerular activity pattern encoding odor identity.

### Pulses in odor concentration

Similar to the ON and OFF ORNs (Burgstaller and Tichy 2011, 2012; Hellwig and Tichy 2016), a group of ON neurons is active throughout the concentration pulse and a group of OFF neurons throughout the odor-free interval. The direct transfer of the presence and absence of the odor pulse into excitatory neural signals enables encoding pulse rates without explicit knowledge of time. Furthermore, the pulse onset or the jump in concentration and pulse offset or the drop in concentration are accentuated by a phasic or phasic-tonic activation of a subset of ON and OFF neurons. Thus, AL neurons convey information about the arrival, duration, and spacing of odor pulses by excitation, as well as the amplitudes of both concentration jumps and drops. However, a group of ON neurons implements fairly early in the olfactory pathway a concentration-invariant representation of lemon-oil odor pulses.

All ON and OFF neurons are active across the entire concentration range (5 to 95%). Therefore, the number of neurons participating in the response to concentration pulses remains constant when pulse concentration changes, but their total activity changes. While the magnitude of the responses of single neurons is proportional to the magnitude of the concentration pulses, indicating the ability to convey information on pulse concentration, the cumulative concentration–response functions, in contrast, reveal no dependence of the total neural activity on pulse concentration. Thus, the pooled responses of the ON neurons provide a concentration-invariant representation of the onset and duration of the odor pulse, and the pooled responses of the OFF neurons represent pulse offset independently of the drop concentration. This independence with respect to concentration does not reflect the variance in the steepness of the slopes of individual concentration–response functions. Rather, it is due to the variance in the response magnitude or the heights of the functions. Importantly, when the functions are scaled on their maximum responses, the cumulative linear functions depend on pulse concentration. Pooling may occur by convergence of the axon of a large number of ON ORNs onto a few ON neurons and by convergence of a large number of OFF ORNs onto a few OFF neurons. Therefore, when the actual responses are pooled, pulse onset and pulse offset are signaled, but not the amplitude of the concentration pulse. By pooling normalized responses, information about pulse amplitude is maintained.

In locusts, the summed responses of 110 PNs to 3 aliphatic alcohols varied little with odor and concentration, and pooling the responses of this population of PNs produced mean responses that were concentration-invariant in a certain range of concentration pulses (Stopfer et al. 2003). In the cockroach, single AL ON-neurons maintains an invariant representation of the food odor over the whole range of concentration pulses, providing the basis for encoding pulse identity independently of pulse concentration. In comparison, the antagonistic concentration-dependent ON and OFF responses improve the efficiency of encoding spatio-temporal concentration changes when tracking an odor plume to the source, moving the antennae and timing these movements. A neural substrate for encoding the presence and loss of the pheromone signal (stimulus On and Off) has been described in the moth *Agrotis ipsilon* (Martinez et al. 2013). PNs in the macroglomerular complex of the moth’s antennal lobe generate a multiphasic response pattern to pheromone pulses. A phasic burst of impulses after stimulus onset is followed by a silent period referred to as inhibition and a long tonic discharge after the termination of the pulse. The strength of the On and Off responses increased with pulse concentration; this also applies for the on-duration but not the inhibitory interval. Robotic experiments demonstrate that the On/Off activity pattern of a model neuron trigger an upwind surge (On response) and crosswind casting (Off response) maneuvers, enabling a cyborg navigation in a pheromone plume and successful localization of the pheromone source.

### Oscillations in odor concentration

All ON and OFF neurons responding to concentration pulses are also activated by oscillating concentration changes. Two groups of ON neurons can be distinguished, based on their temporal response profiles during the up cycles. In the first group, the response profile changes with the duration of the oscillation period: during brief periods (3 and 6 s), the discharge is continuous and smoothly modulated by the changing concentration, but during long periods (60 and 120 s), a phasic activity is superimposed on irregular but continuous discharge rates at values above the noise level, preventing adaptation. In the second group, phasic-tonic discharge rates occur in response to the up cycles of both brief and long oscillation periods. All OFF neurons show a phasic-tonic response during the down cycles, independently of the period duration. However, neurons with different response sensitivities do occur. As the concentration time courses are precisely quantified, these variations can virtually not result from concentration variations.

The oscillating discharge rates of ON and OFF neurons tended not to be in phase with the oscillating instantaneous concentrations, but in advance of them, and furthermore, they tended not to be in phase with the oscillating rates of change either, but behind them. They are intermediary, if not invariably, between the instantaneous concentration and its rate of change, similar to the ON and OFF ORNs (Burgstaller and Tichy 2021). The phase relationship results in a double dependence of the ON and OFF neuron responses to the instantaneous concentration and its rate of change which becomes apparent when impulse frequency is plotted as a function of the two parameters. Impulse frequency of the ON neuron is high when the instantaneous concentration is high and higher still the faster the concentration is rising through these high values, and low when the concentration is falling and lower still the faster concentration is falling, and in between when the concentration is not changing. In contrast, impulse frequency of the OFF neuron is high when the instantaneous concentration is low and higher still the faster the concentration is falling through these low values, and low when the concentration is rising and lower still the faster concentration is rising, and in between when the concentration is not changing. In this way, the effect of concentration is gradually reinforced by the rate of change. The reinforcing effect of the rate of change corresponds with the duration of the oscillation period, indicating that temporally fluctuating odor concentrations initiate processes to regulate the gain and dynamics of the neurons responses maintaining selectivity for input features. The ON and OFF neurons are separable into two subgroups according the degree to which temporal modulations of the odor signal are processed: robust gain control which highlights adjustments in a narrowly focused manner, and variable gain control which acts in a broad, context dependent manner.

Robust gain control is present when during an oscillation period the impulse frequency varies with the instantaneous concentration and its rate of change, but the gain for the two parameters remains fairly constant when the rate of change is varied by changing the period duration. This means, however, that higher rates due to reducing the oscillation period or slower rates due to extending the oscillation period have no decisive effect on the phase relationship between impulse frequency, instantaneous concentration and the rate of change. Hence, the slopes of the regression planes are almost invariant to the oscillation period (Fig. 7c, d; OFF-neuron 3, ON-neuron 4). Robust gain control emphasizes instantaneous concentration at the expense of concentration rates and assures that the neurons activity patterns for encoding odor identity is stable across fluctuations in the concentration rate.

In the case of variable gain control, the impulse frequency varies with the instantaneous concentration and its rate of change, but in contrast to robust gain control, the phase of impulse frequency shifts between the instantaneous concentration and its rate of change depending on the duration of the oscillation period (Fig. 7a, b; ON-neurons 1, 2). This means, that higher rates during briefer periods shift the ON-neurons’ response maxima toward the maxima of the instantaneous concentration, and lower rates during longer periods shifts them toward the maxima of the rate of change. Based on the negative concentration coefficient of the OFF-neurons responses, a decrease in the period duration shifts the response maxima toward the minima of the instantaneous concentration, and an increase toward the minima of the rate of change.

The phase shift appears as a trade-off between the gain values of the two parameters which favors during brief periods a higher sensitivity for the instantaneous concentration at the expense of the sensitivity for the rate of change, and during long periods, a higher sensitivity for the rate of change at the expense of the sensitivity for the instantaneous concentration. During slow oscillations with long periods, the improved gain for the rate of change enables ON and OFF neurons not only to keep up with both rapid and slow fluctuations in odor concentration, but also to balance—from instant to instant—their sensitivity according to the rate at which concentration changes. During rapid oscillations, in contrast, gain control prevents the ON and OFF neurons from reaching saturation and decreases the sensitivity for concentration increments and decrements. During slow oscillations, cockroaches need to determine whether the discharge rate is changing at all. Because of the high gain for low rates of change, the neurons are best suited for detecting and processing slow concentration changes, even if they maintain for several minutes without changing directions. Gain control provides high precision for slow rates when this is vital, without narrowing the detectable and usable concentration range during orientation and without expanding the response scale.

Variable gain control in the ON and OFF neurons involves moderate or strong dependence on the duration of the oscillation period. Neurons with strong dependence show an extreme phase shift of the frequency maxima either close to the maxima of the instantaneous concentration or close to the maxima of the rate of change. The result is a higher gain for the component of the stimulus that is approached ever more closely by varying the period duration and a weaker or no gain for the other component of the stimulus that becomes increasingly distanced. In this way, the gain for one component of the fluctuating odor concentration is intensified and the gain for the other component is attenuated. Variable gain control with a strong dependence of gain on the period duration adjusts sensitivity to a range of rates of concentration changes. This may more efficiently represent the fluctuating concentration pattern in the environmental context in which such concentration changes occur.

Note that both the robust and variable gain control make the ON and OFF AL neurons more informative than the variable gain control of the ON and OFF ORNs. One might expect that cockroaches primarily use an increase in impulse frequency as a setting with which to identify odor quality. The cue will be what odor elicits the increase in impulse frequency. Robust gain control is perfectly suited for this task by neglecting the concentration of odor pulses or variations in rate of change due to variations in the duration of the fluctuation period. Nonetheless, orientation to an odor source would benefit from extracting guidance cues from concentration fluctuations in a turbulent plume. Variable gain control emphasizes detecting the rate of change on different time scales and maintains efficient encoding of fluctuating concentration changes even when the duration of the oscillation period greatly varies. The existence of two gain control mechanisms indicate that key aspects of the odor stimulus are extracted and processed separately in two parallel systems.

Several studies conducted with different techniques have shown that gain control achieved by the AL network may be thought of as an amplifier of neural communication, supporting a certain degree of concentration invariant odor representation (Olsen and Wilson 2008; Olsen et al. 2010; Zhang et al. 2013; Marachlian et al. 2019; Gjorgjieva et al. 2019; Martelli and Storace 2021). In *Drosophila*, gain control is mediated by lateral interactions across many olfactory glomeruli and helps to equalize the population response of projection neurons (PNs). Following brief concentration pulses, gain control amplifies weak ORN inputs and attenuates strong ORN inputs. An increased input with increasing pulse concentration is due to the convergence of numerous axons of ORNs into a single glomerulus, where they make excitatory synaptic contacts with the apical dendrites of a small number of second-order PNs. In addition to the excitatory sensory input from ORNs, each PN receives inhibitory synaptic input (lateral inhibition) from neighboring glomeruli via local interneurons. They reduce the activity of PNs when other glomeruli are co-activated by the same strong concentration pulse. Lateral inhibition provides negative feedback to PNs and normalizes the total activity patterns of the PN population, leading to a firing rate that is mostly independent of odor concentration (Sachse and Galizia 2003; Asahina et al. 2009; Olsen and Wilson 2008; Olsen et al. 2010; Wilson 2013; Hong and Wilson 2015). It would be interesting to know whether slow and continuous rates of concentration changes also trigger lateral inhibition of PNs by the local-neuron’s negative feedback.

In *Drosophila*, a weak input discharge arises when few, yet very sensitive ORNs respond to low amplitude, pulse-like concentration changes. Conversely, a strong input discharge stands for the response of a higher number of ORNs to large amplitude, pulse-like concentration changes. In the cockroach, on the other hand, a weak input discharge is the response of a given number of highly sensitive ON and OFF ORNs to slowly fluctuating changes in concentration across the entire concentration range, and accordingly, a strong input discharge is the response of the same number of highly sensitive ON and OFF ORNs to rapidly fluctuating concentration changes across the same concentration range. Gain control in the cockroach adjusts sensitivity according to the prevailing fluctuations in odor concentration signaled by the ON and OFF ORNs, but gain control in *Drosophila* provides an average of the convergent input of many ORNs. However, gain control that matches the input–output function to the distribution of the fluctuating odor concentration encountered during plume tracking cannot be achieved by averaging the inputs of many ORNs converging at the level of the AL. Furthermore, a subset of ON neurons’ responses implement a concentration-invariant representation of the odor stimulus. Therefore, normalization of AL responses due to lateral inhibition is not required.

Gain control at the level of PNs can reasonably get involved in adjusting sensitivity based on a measure of the temporal dynamics of odor samples collected at a local antennal region. Efficient encoding, however, requires that gain be controlled as rapidly as concentration changes. Gain controls for the rate of concentration change that are engaged following a change in the mean input across numerous ORNs are probably not controlled by the mean rate of concentration change averaged across these ORNs. Instead, they will be controlled by a neural signal that depends directly on the rate of change, for example, the input of ORNs in the same spatial receptive field. Noise in the mean discharge rates of ORNs due to slowly changing concentrations may produce noise in the signal controlling gain. This noise could act as a real change in the mean concentration rate and hence produces changes in gain. This makes it advantageous to adjust sensitivity to the rate of change before combining the convergent ORN responses. Gain controls that extract the relevant information from single ORNs go beyond merely contributing substantially to the overall control of the gain of ORN-mediated responses. Evaluating the concentration rate in the single ORN is less noisy than evaluating the mean and less difficult on the time scale of the concentration change. The more the immediate gain is controlled, the more precisely sensitivity is adjusted to the prevailing rate of concentration change.

### Contribution to orientation

The impulse frequency of the ON and OFF neurons depends on the direction and extent of concentration changes, as well as on the instantaneous concentration and its rate of change. Individual responses are, therefore, ambiguous, but this does not necessarily mean that the neurons are unable to supply the central nervous system with useful information on these parameters. Rapid excitation signals that a transient concentration change is a component of the stimulus and slow excitation indicates that a concentration fluctuation enters as a component. When the elicited impulse frequency is limited to the lower portion of the frequency scale, low-amplitude transient concentration changes or slowly fluctuating changes with long periods are stimulus components. When the stimulus forces impulse frequency to the upper portion of the frequency scale, then any frequency represents a whole set of combinations of large-amplitude transient concentration changes and high rates of change during brief periods. Nevertheless, smooth impulse response functions of an ON neuron means a brief upward concentration wave and irregular responses a long upward wave.

The possibility for a somewhat finer distinction between oscillations of different periods seems to be provided by taking several neurons individually and then compare their responses. By observing which neuron is discharging at its maximum frequency range and whether the discharge is continuous and smooth or phasic and superimposed on irregular continuous discharge rates with the maximum in the upper range (or alternatively phasic or irregular and with a maximum in the lower range), concentration pulses and concentration fluctuations may be distinguished. This process supposes that enough neurons exhibit such a maximum so as to cover the range of concentrations and rates of change. The present study indicates large variation in the individual responses within the ON and OFF neuron groups which are not errors in olfactory coding. In fact, that variability allows an instantaneous analysis of the spatial distribution of odor concentration and its rate of change by different neurons in the cockroach’s olfactory system. Robust gain control facilitates this task be increasing sensitivity for the instantaneous concentration and by neglecting variations in rate of change due variations in the duration of the oscillation period.

Within a given oscillation period, individual neurons have a characteristic impulse frequency versus instantaneous concentration and rate of change 3-D surface in which each frequency value corresponds to a certain combination of the two components of the fluctuating odor stimulus. In so far as the slopes of their regression planes differ from neuron to neuron, the characteristic response surfaces of the different neurons also differ. Handling the output of different neurons simultaneously should reduce the number of possible combinations of the stimulus components eliciting a given frequency value or even the frequency maximum. A single common point of intersection should emerge from simultaneously considering three or more neurons from different subsets. Each point of intersection and frequency maximum arising therefrom may correspond to a given rate of change or pulse slope, thereby creating a sequence of slopes with increasing steepness. As the number of neurons increases, errors from random response variation should become smaller. Let us suppose that a number of neurons have maximum response to fluctuating concentration changes in the physiological range of rates of change between brief and long durations. Then, the CNS could be so circuited as to force the cockroach to seek—on its way to the odor source—surroundings, where the sequence of frequency maxima from the simultaneous evaluation of ON neuron sets best occur. If departure from these frequency sequences occurs, then the OFF neurons would be very effective at falling concentration because of their high sensitivity to decreasing instantaneous concentration and rate of decrease. Tuning neurons to the different temporal concentration changes or “pulse slopes” would enable an accurate extraction of the spatial information in an odor plume by different neurons and would be advantageous in determining the direction and distance to the odor source. Variable gain control emphasizes the detection of the rate of change on different time scales and maintains efficient encoding of the fluctuating concentration pattern when the duration of the oscillation period varies. Robust gain control does not require the recognition of the duration of the period or the “meaning” of the rate of change contained in the input signal to precede processing, but variable gain control requires a degree of recognition to concur with the act of processing. Variable gain control is governed by factors contained in the rate of change, such as “context”. The different gain control properties indicates that the complexity of the odor signal leads to labor division: key aspects of the signal are extracted and processed separately in two parallel systems that operate simultaneously rather than sequentially.

Many researchers have examined robotic plume tracking inspired by animals and in particular insects. The conceptual model of the Braitenberg vehicle (1984) has been used to characterize the cockroach’s performance to successfully localize the odor source of a steady state odor plume with a smooth, continuous gradient toward the source. It has also been used for a non-steady state dispersion generating a fluctuating plume with dynamic concentration changes (Pequeno-Zurro et al. 2018, 2020, 2021; Shaikh and Rano 2020). The vehicle model has two identical chemical sensors, symmetrically placed at the front of the vehicle. Those sensors generate positive chemotaxis through inhibitory ipsilateral connections between the sensors and the two wheels. In its simple form, the vehicle uses only instantaneous stimulus sampling and does not account for the temporal dynamics of concentration. The vehicle model inspired by the ON and OFF ORNs on the cockroach antennae incorporates a linear weighted combination of instantaneous stimulus with temporal stimulus dynamics within the sensorimotor couplings. The vehicle without temporal dynamics odor processing works best in environments with a spatial concentration gradient: it oscillates around the air flow direction and eventually directly faces the source. The vehicle that includes the temporal dynamics of odor processing in the sensorimotor connections reduces the oscillations in the vehicle’s trajectories. The trajectories, however, are longer and seem to oscillate with a larger amplitude than the trajectories of the vehicle without temporal dynamics of odor processing (Pequeno-Zurro et al. 2018, 2020, 2021; Shaikh and Ignacio 2020). Interestingly, the male cockroach utilizes antenna-topic mapping to obtain information about the spatial distribution of the female pheromone plume, i.e., the space along the antenna is maintained as space within the macroglomerulus of the AL (Nishino et al. 2018; Paoli et al. 2020). Several key interneurons for sensing pheromones have been identified, each of them tuned to receive signals only from a certain portion of the cockroach's antenna. Assuming that the spatial arrangement of the ON and OFF ORNs generates a corresponding spatial representation in the AL, the cockroach would have access to the spatial distribution of the temporal dynamics of food odor concentration on the antenna. Using information about the rate at which concentration changes on limited parts of the antenna next to each other could potentially shorten the trajectories of the dynamic vehicle model even further.

## Conclusions

The ON and OFF AL neurons provide excitatory responses to increments and decrements in the concentration of a food odor. This enables rapid information transfer for both stimulus onset and offset, improves the temporal contrast, and signals directly the duration of the odor stimulus and the inter-stimulus interval. Some of the ON neurons exhibit concentration-invariant responses over the entire range of odor concentrations, enabling them to focus on representing or extracting a special aspect of the odor stimulus, such as odor identity. The ON and OFF responses to rising and falling concentration depend on the rate with which concentration changes, according to variations in the duration of the oscillation period. This provides perfect adaptation for efficiently encode the fine temporal dynamics of periodic fluctuations in odor concentration. The information on the different stimulus features is encoded by individual members of the ON and OFF neurons with different degrees of sensitivity and cannot be relayed by simply summing or averaging the responses of all neurons engaged by concentration changes. This is because they differ in their selective representation of a specific stimulus features. A more efficient form of pooling would give a low weight to poor-quality responses of relatively low sensitivity and temporal resolution and a stronger weight to high-quality responses. When the weighting is optimal rather than equal, no useful information would be wasted. A deeper level of analysis is obtained by defining the neural representation of physical features of fluctuating odor concentration that relates to the onset slopes of the changing concentration or the rate of concentration increase. Variations in the response properties of individual ON and OFF neurons could allow the olfactory system of a plume tracking cockroach to detect concentration changes at different rates using an across-fiber pattern.

## Data Availability

The datasets generated and analysed during the current study are available from the corresponding author on reasonable request.
